# Rationally designed Human Cytomegalovirus gB nanoparticle vaccine with improved immunogenicity

**DOI:** 10.1371/journal.ppat.1009169

**Published:** 2020-12-28

**Authors:** Michela Perotti, Jessica Marcandalli, Davide Demurtas, Federica Sallusto, Laurent Perez

**Affiliations:** 1 Institute for Research in Biomedicine, Università della Svizzera italiana, faculty of Biomedical Sciences, Bellinzona, Switzerland; 2 Institute of Microbiology, ETH Zürich, Zürich, Switzerland; 3 BioEM Facility, School of Life Sciences, Swiss Federal Institute of Technology Lausanne (EPFL), Lausanne, Switzerland; 4 University of Lausanne (UNIL), Lausanne University Hospital (CHUV), Department of Medicine, Division of Immunology and Allergy, Center for Human Immunology (CHIL), Lausanne, Switzerland; University of Minnesota, UNITED STATES

## Abstract

Human cytomegalovirus (HCMV) is the primary viral cause of congenital birth defects and causes significant morbidity and mortality in immune-suppressed transplant recipients. Despite considerable efforts in vaccine development, HCMV infection still represents an unmet clinical need. In recent phase II trials, a MF59-adjuvanted gB vaccine showed only modest efficacy in preventing infection. These findings might be attributed to low level of antibodies (Abs) with a neutralizing activity induced by this vaccine. Here, we analyzed the immunogenicity of each gB antigenic domain (AD) and demonstrated that domain I of gB (AD5) is the main target of HCMV neutralizing antibodies. Furthermore, we designed, characterized and evaluated immunogenic responses to two different nanoparticles displaying a trimeric AD5 antigen. We showed that mice immunization with nanoparticles induces sera neutralization titers up to 100-fold higher compared to those obtained with the gB extracellular domain (gB_ECD_). Collectively, these results illustrate with a medically relevant example the advantages of using a general approach combining antigen discovery, protein engineering and scaffold presentation for modern development of subunit vaccines against complex pathogens.

## Author summary

Human cytomegalovirus (HCMV) infection is the foremost viral cause of congenital birth defects. Attempts to vaccinate against HCMV brought moderate, though not sufficient, reduction in infection. Moreover, phase II clinical trial with gB formulated in MF59 adjuvant did not induce a neutralizing antibody response. Here, we identify the antigenic domain 5 (AD5) of gB as the target of most neutralizing antibodies. Moreover, functionalization of AD5 on self-assembling nanoparticles leads to gB vaccine candidates generating high antibody binding and neutralizing titers. Interestingly, it was recently reported that most antibodies targeting AD5 could inhibit cell fusion, a discovery of interest as HCMV is thought to disseminate within the host primarily through direct cell-to-cell spreading. Our data not only provide new vaccine candidates but may also have important implications for understanding cellular fusion promoted by HCMV.

## Introduction

Human cytomegalovirus (HCMV) is a ubiquitously distributed member of the *Herpesviridae* family that establishes lifelong infection in human [1]. HCMV estimated seroprevalence ranges from 50% to 100% in the human adult population worldwide [2]. Primary infection during pregnancy is the most frequent cause of congenital birth defects with 20% of infants infected in utero developing long-term sequelae including neurological damage, growth retardation, hearing loss and microcephaly [3]. Moreover, HCMV is responsible for high morbidity and mortality in immunocompromised patients such as solid organ (SOT) and hematopoietic stem cell (HSCT) transplant recipients [4,5]. Frequent use of current antiviral therapies can have toxic effects and potentially lead to HCMV escape mutants [6]. Furthermore, infusion with hyperimmune globulins to control viremia is not efficient [7]. Given the severity and importance of this virus, “The National Vaccine Advisory Committee” in the US classified the generation of a vaccine against HCMV as a top priority since 2004 [8–10]. The major target populations for vaccination are seronegative women of childbearing age and, among the others, seronegative patients awaiting organ transplantation, who are at risk for life-threatening HCMV disease. HCMV cellular entry is analogous to the one employed by other members of the *Herpesviridae* family as reviewed elsewhere [11–13] and requires different glycoprotein complexes present on the virion envelope for viral binding and cellular entry into the host cell. Among them, the homotrimeric glycoprotein B (gB) has been reported as an essential factor for cellular entry, being the direct mediator of HCMV fusion within all targeted host cell membranes [14,15]. A subunit vaccine based on gB adjuvanted with MF59 showed a modest efficacy in limiting primary viral infection in seronegative women and reducing duration of viremia in transplant recipients [16–18]. These results might be explained by the finding that most antibodies induced by the vaccine lack a viral-neutralizing activity [16,17]. Nonetheless, the gB glycoprotein has been recently demonstrated to be the target of antibodies eliciting a strong antibody-dependent cell mediated cytotoxicity (ADCC) in patients after SOT [19]. Although the recombinant gB based vaccine was not able to reach a full protection, a partial efficacy in sterilizing immunity was observed, prompting the possibility of developing a modified vaccine with an increased efficacy [20–22].

The gB homotrimer contains five known antibody target sites (antigenic domains: ADs) [23,24]. AD1 relates to the structural domain IV and is an immune-dominant region of gB [25]. AD2 corresponds to the N-terminal fragment and was originally defined between residues 27 and 84 [26,27]. AD3 is the cytosolic domain of gB and, unsurprisingly, it is known to generate exclusively non-neutralizing antibodies [28,29]. AD4 is a discontinuous epitope defined by the structural domain II [30–32]. Finally, AD5 corresponds to the structural domain I and is the target of neutralizing antibodies (nAbs) [30]. Recent works have potentially improved the efficacy of the gB vaccine, by using mRNA encoding only the gB extracellular domain that excludes AD3 (gB_ECD_). This vaccine candidate elicited antibody responses with great durability and breadth [20]. To date, immunogenicity of the individual ADs has been poorly characterized, even though this information is crucial to guide vaccine design. Moreover, it is well known that presentation of multiple copies of an antigen in a repetitive array such as nanoparticle or virus like particles (VLP) drives a more robust humoral immune response compared to its soluble (unconjugated) counterpart [33]. Several technologies have been explored to generate nanoparticles for antigen display [34], and, among them, self-assembling proteins were proven to be a powerful platform for multivalent antigen presentation, as they can form highly ordered, monodisperse structures [35]. Recently, several self-assembling proteins such as ferritin [36] and de novo computationally designed self-assembling proteins [37] have been successfully used as scaffolds to present complex glycoprotein antigens derived from influenza hemagglutinin [36] and the fusion protein of the Respiratory Syncytial virus (RSV) [37]. In all cases, immunogenicity of the antigen has been increased by multivalent presentation.

In this study, we report a systematic analysis of gB ADs immunogenicity based on mice immunization, which indicates that AD5 is the only AD that triggers nAbs against HCMV gB in absence of complement. Based on this discovery, we developed two-multimeric gB immunogens that fuse a newly designed trimeric AD5 (trAD5) to self-assembling I5350 and Ferritin nanoparticles. Our results demonstrate that mice immunization with trAD5-nanoparticles generates antibodies that are 50 to 100-fold higher in binding and neutralizing properties compared to those induced by the gB_ECD_ immunogen.

## Results

### Generation of recombinant gB_ECD_ and its different ADs

**Fig 1:**
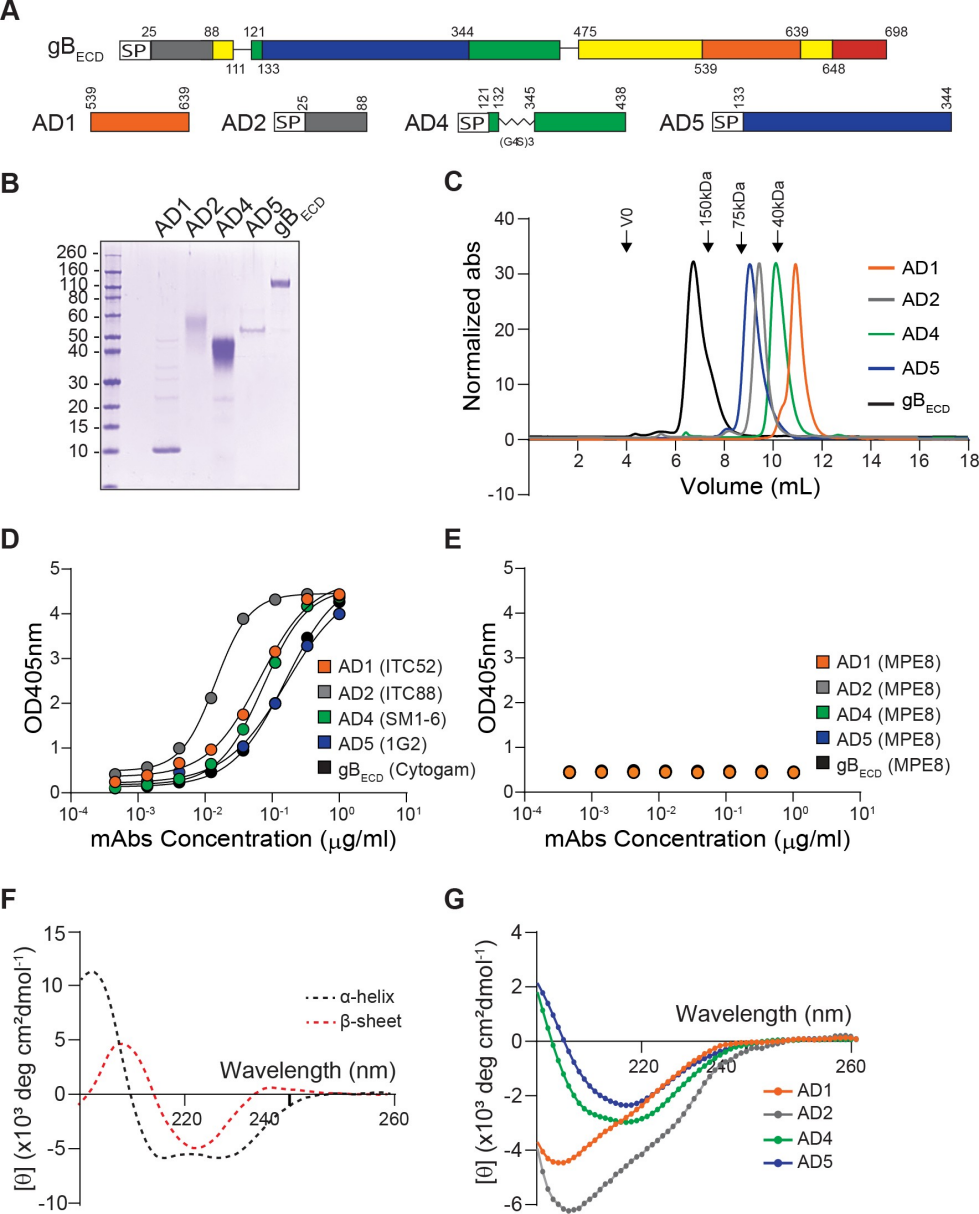
HCMV gB antigens design and biochemical analysis. (**A**) Graphical representation of gB extracellular domain (gB_ECD_) and the antigenic domains (ADs). AD1 is in orange, AD2 in grey, AD4 in green, AD5 in blue and the core domain is in yellow. SP indicates the interleukin 2 (IL2) signal peptide. (**B**) Reducing SDS-PAGE of purified immunogens. Molecular weight marker is indicated in kilodalton (kDa). (**C**) HPLC-Size Exclusion Chromatography analysis of the gB_ECD_ and ADs showing that they elute as a homogenous and monodisperse product. (**D**) Domain-specific ELISA with mAbs in serial dilution binding to the different antigens used for immunization. Antigens were coated at 3 μg/ml. (**E**) Control ELISA with MPE8 in serial dilution with the different antigens used for immunization coated at 3 μg/ml. (**F**) Far-UV spectra for two specific peptides containing only α-helix or β-Strands (0.5 mg/mL) were recorded over the same wavelength. Spectra are the average of three repeats and blank is subtracted. (**G**) Far-UV spectra for gB_ECD_ and ADs (0.5 mg/mL) were recorded over the wavelength range of 180–260 nm. Each panel shows one representative result of three independent experiments.

Previous studies evaluating gB and ADs immunogenicity relied mainly on the observed potency of monoclonal antibodies (mAbs) isolated from seropositive donors [30]. To obtain detailed information on the humoral immune response generated by each AD, we decided to immunize mice with each of them independently. The entire gB extracellular domain (gB_ECD_) was generated as previously described [23] and used as control. The gB domains AD1, AD2, AD4 and AD5 were based on the gB crystal structure (PDB ID: 5C6T) of the HCMV Merlin strain. AD3 domain was not produced in this study since it was already shown to be unnecessary for a potent antibody response [20]. To produce a soluble gB_ECD_, we used residues 25–698 with a mutated Furin cleavage site followed at the C terminus by a TEV protease site, 6-histidines and a tandem Strep-tag for purification purpose. The antigens produced correspond to AD1 (residues 539–639), AD2 (residues 25–88) and AD5 (residues 133–344). Expression and production of AD4, which is composed of two discontinuous domains (121–132 and 345–438) required the connection of the latter by a flexible 15 amino acids linker (Gly_4_Ser)_3_ (**Fig 1A**). All constructs carried a signal peptide (IL2 sequence) for secretion purpose, with the exception of AD1 produced in bacteria. Purified HCMV gB_ECD_ and ADs were analyzed for purity by SDS-PAGE (**Fig 1B)**. We observed that gB_ECD,_ AD1 and AD5 show the expected molecular weight (**Fig 1B)**, while AD2 and AD4 migrated at a higher molecular weight than expected and appeared as a smear, confirming that both of these domains are heavily glycosylated [23]. Next, to assess the homogeneity and the absence of aggregation in the purified antigens, we analyzed them by HPLC size exclusion chromatography (HPLC-SEC) (**Fig 1C**). The latter showed a single peak at the expected molecular weight for each of them: 160kDa for gB_ECD_, 10 kDa for AD1, 60 kDa for AD2 glycosylated, 40 kDa for AD4 glycosylated and 55 kDa for AD5. To address the correct folding of each antigen, we performed ELISA assays using a panel of human mAbs specific for gB and recognizing conformational epitopes, namely ITC52 (AD1), ITC88 (AD2), SM1-6 (AD4) and 1G2 (AD5) (**Fig 1D**) [30,31,38,39]. We observed a strong reactivity of the aforementioned mAbs to their respective target, confirming that the immunogens we produced have the same folding either separately or in the whole gB protein. Of note, assay specificity was confirmed by probing gB_ECD_ and ADs with the anti-F RSV mAb MPE8 as negative control (**Fig 1E**) [37]. Moreover, gB antibodies specificities were assessed by showing an absence of signal, when they are used on plates coated with F-RSV in postfusion conformation [40] (**S1 Fig**). Furthermore, to validate antigen folding, we performed a secondary structure analysis using Circular Dichroism (CD) spectroscopy. The amides present in folded part of the protein strongly absorb circularly polarized light and undergo varying extents of n→π* and π→π* transitions for a given wavelength [41]. For instance, pure α-helical structure exhibits a characteristic double minimum at 208 nm and 222 nm as seen with the well-defined synthetic peptides, forming alpha helices (PDB ID: 1AL1). Similarly, β-sheet structure exhibits a characteristic minimum at 215 nm as seen with a protein containing β-strands (PDB ID: 6E5C) (**Fig 1F)** [42,43]. We analyzed the AD1 (domain IV) and found that it is predominantly composed of β-sheets, while AD4 and AD5, structural domains II and I respectively, adopt a typical Pleckstrin Homology (*PH*)-domain-like fold, characterized by seven stranded antiparallel β-sheet and a C-terminal α-helix [44]. AD2, whose 3D structure is unknown, appears to be composed of random coils and β-sheet. The analysis of our acquired spectra revealed a secondary structural composition that is in agreement with known and reported structural data for gB_ECD_ [23,24] (**Figs 1G** and **S2**). Taken together, our data indicate that gB_ECD_ and the ADs are pure, correctly folded and non-aggregated, also no mass spectrometry analysis was performed on the sample.

### AD5 is the main target of HCMV gB neutralizing antibodies

**Fig 2:**
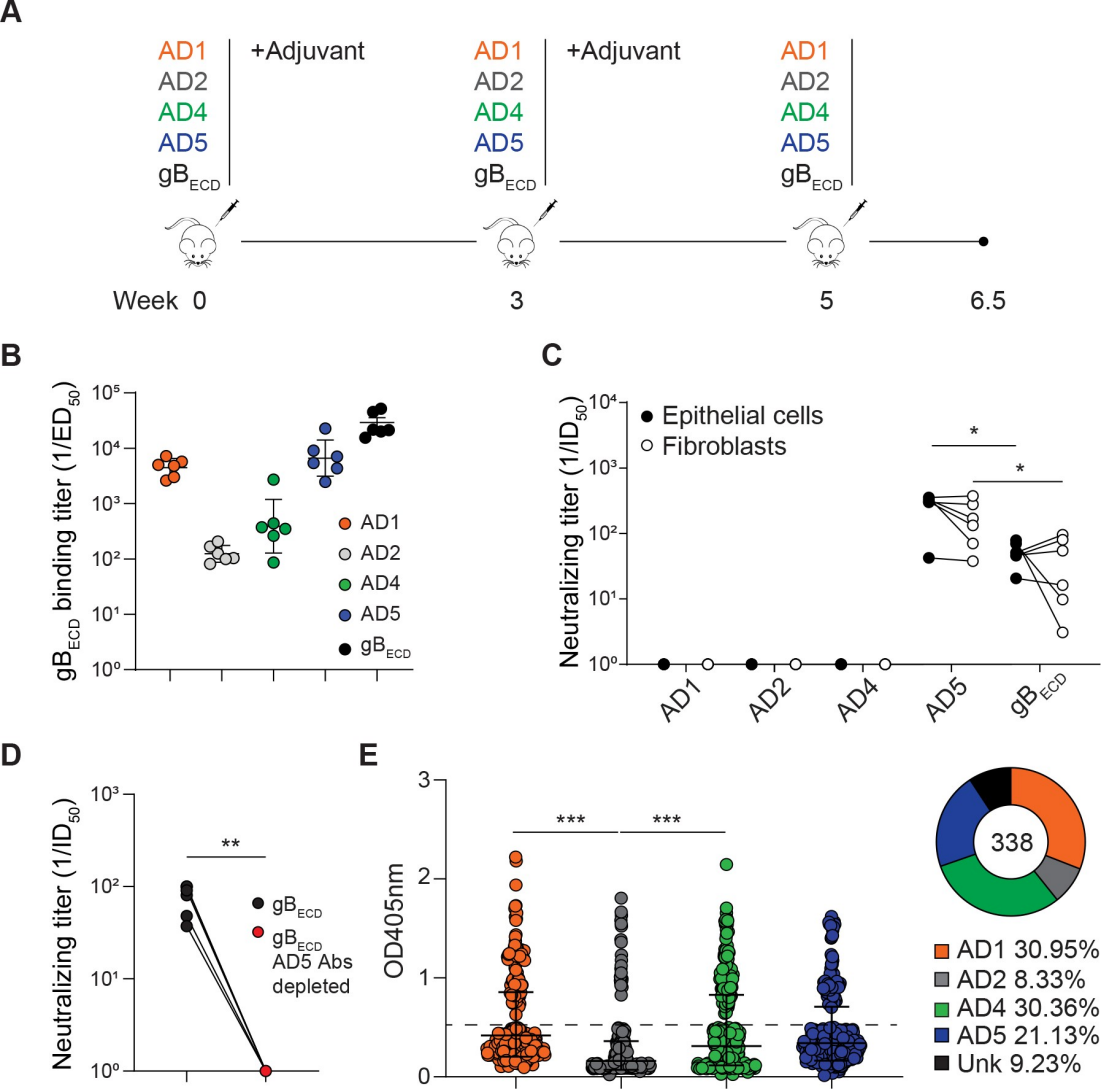
Serum binding and neutralizing titers in mice immunized with gB_ECD_ and ADs. (**A**) Schematic representation of mice immunization schedule. BALB/c mice were immunized with a total protein dose of 5 μg formulated in PBS in a 1:1 ratio with Ribi Adjuvant. Mice were immunized s.c. on week 0 and 3 with adjuvant, followed by a boost without adjuvant on week 5. Mice were bled on week 6.5. (**B**) Inverse IgG serum antibody binding titers (1/ED_50_) to gB_ECD_. (**C**) Inverse IgG serum antibody neutralizing titers (1/ID_50_) measured on epithelial cells (ARPE-19, black circles) or fibroblasts (MRC-9, white circles). (**D**) Inverse serum antibody neutralizing titers after depletion of anti-AD5 antibodies. (**E**) *B cells* culture *supernatant* specific for gB_ECD_ were tested on each antigenic domain and their distribution is represented as a pie chart. Each panel showed one representative result of three independent experiments. Significance was calculated using Kruskal-Wallis + post hoc Mann-Whitney U test. Marked with (*) for p < 0.05, (**) for p < 0.01, and (***) for p < 0.001. Plotted are geometric means and Error bars show SD of the geometric mean values.

To investigate the contribution of each AD in the humoral immunogenicity of gB, we immunized mice with the soluble recombinant gB_ECD_ as control, AD1, AD2, AD4 or AD5 (Merlin strain). BALB/c mice were immunized by subcutaneous (s.c) injection of 5 μg of antigens formulated in Ribi Adjuvant (priming and boost one) [45]. The Ribi adjuvant is a stable oil-in-water emulsion and was chosen for its ability to preserve native protein conformations [46]. The immunization protocol with the different antigens is shown in **Fig 2A**. Immunization with recombinant gB_ECD_ induced antibodies that bound to gB with an average ED_50_ of 10^4.5^ and neutralized HCMV infection of epithelial cells and fibroblasts with an average ID_50_ of 10^1.7^ and 10^1.6^, respectively (**Fig 2B**), a result in agreement with previous reported data [16,17,21,47]. Immunization with AD1, AD2, AD4 and AD5 also induced antibodies that bound to both gB_ECD_ (ED_50_ of 10^3.7^, 10^2.1^, 10^2.8^ and 10^3.9^ respectively) and to their corresponding immunogen (**Figs 2B** and **S3A**), but not to the F-RSV protein used as control (**S3B Fig)**. Sera analysis revealed that immunization with AD1, AD2 and AD4 did not elicit any detectable antibody titer capable of neutralizing HCMV infection in neither epithelial cells nor fibroblasts in absence of complement (**Fig 2C**), despite the binding titers observed (**Figs 2B and S3A**). These results correlate with previously reported data demonstrating that only few of the anti-AD1, anti-AD2 and AD4-specific antibodies were able to neutralize HCMV *in vitro*, in absence of complement [16,17,30,38,48,49]. Interestingly, AD5 immunization induced antibodies that showed a significantly higher neutralizing activity compared to antibodies generated by gB_ECD_ antigen with an ID_50_ of 10^2.5^ on epithelial cells and 10^2.2^ on fibroblasts (**Fig 2C**). To characterize the extent of the neutralizing anti-AD5 response in mice immunized with gB_ECD_ as immunogen, we depleted the antibodies specific for AD5 from sera of gB_ECD_-immunized mice. Surprisingly, depletion of antibodies binding AD5 dramatically decreased the overall neutralization activity of these sera (**Fig 2D**). This result demonstrates that AD5 is the main target of HCMV gB neutralizing antibodies when gB_ECD_ is used for mice immunization. In a previous study performed in rabbits, it was reported that gB-specific non-neutralizing antibodies were able to acquire protective features in the presence of complement [49]. Therefore, we investigated the neutralization activity of the sera in the presence of 5% complement. We could detect a neutralization titer in presence of complement for gB_ECD_ and AD 5 that were similar to the observed titer without complement (**Fig 2C**). However, with observed neutralization with sera obtained from mice immunized with AD1 (**S3C Fig**) [49]. Surprisingly, the neutralization was observed only on ARPE-19 epithelial cells and not MRC-9 fibroblast (**S3D Fig**). The mechanistic detail behind this observation is unknown, but should be further explored in the future.

To investigate the specificity of the antibodies produced following gB_ECD_ immunization, we collected and cultivated splenocytes from two of the gB_ECD_-immunized mice. Splenocytes were seeded at a density allowing polyclonal stimulation of B cells in presence of CpG synthetic oligonucleotides stimulating murine TLR9. The obtained culture supernatants were tested for gB binding activities by direct ELISA. The percentage of gB specific B cell clones was evaluated to rank from 17.3% to 19.7% of total IgG^+^ B cells. In total, we isolated 338 gB specific clones and decided to analyze the frequency of clones producing antibodies specific for the different ADs. Interestingly, we observed that only a minor fraction of the clones produced gB-specific IgG antibodies against AD2 (8.33%), while most mAbs bound to AD1, AD4 or AD5 (30.95%, 30.36%, and 21.13%, respectively) (**Fig 2E**). Nonetheless, we noted that these results are consistent with data obtained with human immune or vaccines donors [16,30,38] and confirm that the majority of antibodies produced following recombinant gB immunization are not neutralizing and directed towards decoy antigenic domains, such as AD1, AD2 and AD4. Since gB is well conserved at the amino acid sequence level [50], it is tempting to speculate that these results might represent a molecular mechanism at the protein level to evade efficient neutralization of cell free virus [51]. Taken together, our analysis demonstrates that AD5 is able to generate a neutralizing antibody response against HCMV. This interesting result motivates us to functionalize AD5 as a vaccine.

### Design of trimeric AD5 (trAD5) antigen for nanoparticle display

**Fig 3:**
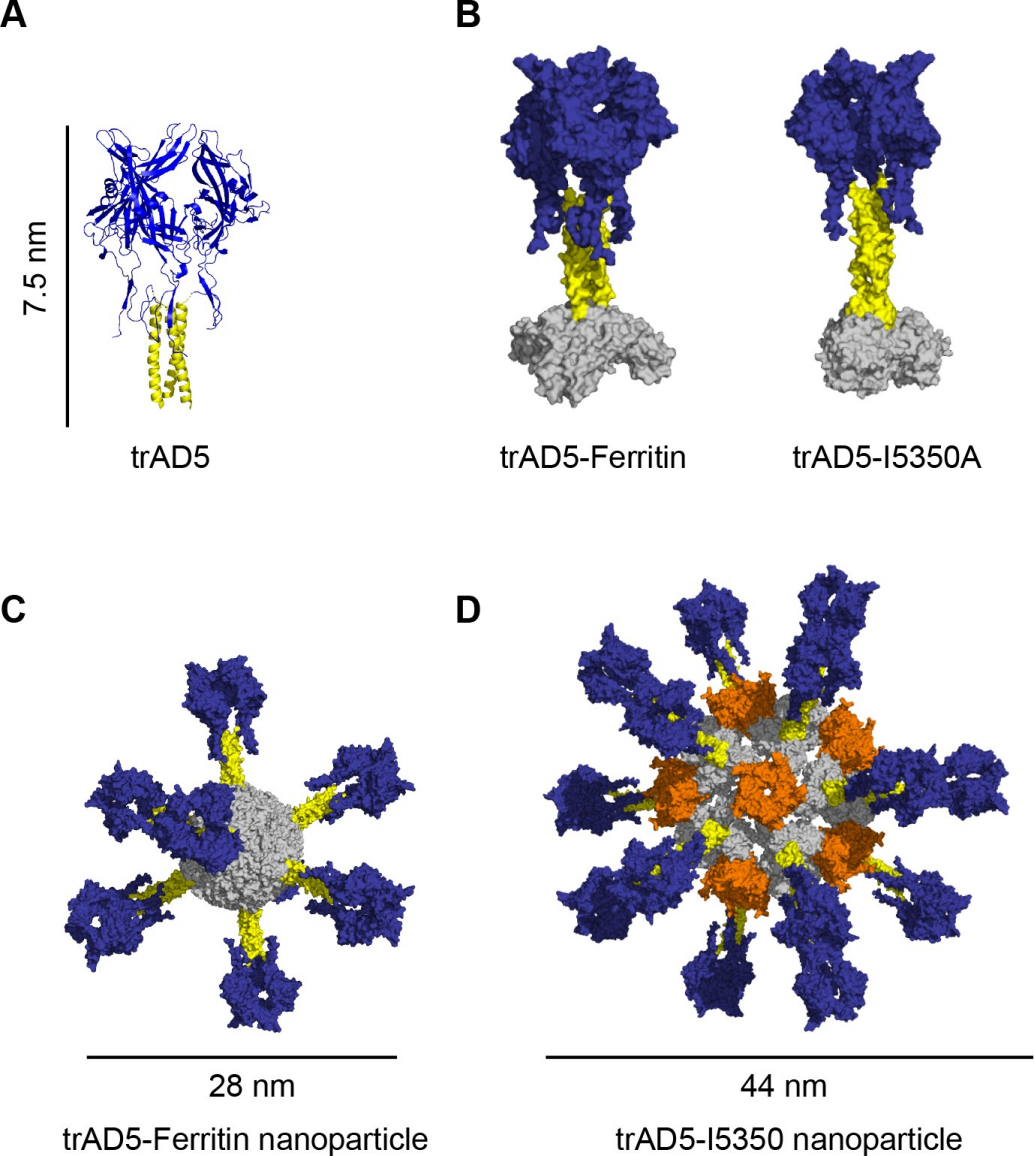
Design of trimeric AD5 (trAD5) and fusion to nanoparticle. (**A**) Schematic representation of the designed trimeric AD5 (trAD5) based on the PDB ID: 5C6T [23]. The C terminus of AD5 (shown in blue) was fused to N terminus of the core domain coiled-coil (shown in yellow) to generate trimeric AD5 (trAD5). (**B**) Computational docking of trAD5 (blue and yellow) to Ferritin and I5350A, both are shown in gray. (**C**) Structural model of trAD5-Ferritin and (**D**) trAD5-I5350. Ferritin nanoparticle displays eight trAD5 and I5350 nanoparticle shows twenty trAD5 fused to I5350A, shown in grey and twelve pentamer of I53-50.4BPT1 shown in orange.

Upon demonstration that AD5 is the main target of HCMV gB neutralizing antibodies, we sought of developing an improved and effective HCMV vaccine based on this domain. Nanoparticles presenting multiple copies of an antigen in a repetitive array [52], have been reported to improve the immunogenicity of the latter. We decided to take advantage of the Ferritin nanoparticle scaffold [36,53] and our recently reported de novo designed protein nanomaterial platform I5350 [37]. The structural characterization of gB indicated that domain III (DIII), or core domain (CD), is a long helix that forms a central triple coiled coil motif within the extracellular domain of the gB trimer [24]. The presence of a threefold symmetry in gB appears as a suitable strategy to dock an engineered AD5 antigen to particles with a C3 symmetry axis. We performed computational modeling to design an AD5 trimer (trAD5), a fusion protein of gB-AD5 with gB-CD. Our construct comprises a direct fusion of AD5 (amino acid sequence 121–347 incorporating 2 extra amino acids from AD4) with a flexible linker to residues 475 to 531 of the core domain (**Fig 3A**) and the modelling was finalized with energy minimization [54]. The designed trAD5 was fused to Ferritin or I5350A chains in presence or absence of the natural trimerization sequence of the T4 Fibritin (foldon) domain [55] (**Fig 3B)**. Self-assembling proteins such as Ferritin or I5350 are able to form nanoparticles with a threefold symmetry axis and display antigens as a repetitive array. This was shown to enhance the likelihood of activating high avidity B cells expressing a BCR specific for the antigen, hence increasing the antibodies and neutralizing titers against HCMV gB [35]. Both fusion constructs ensured a trimeric conformation and the long C-terminal helix prevented steric hindrance with the nanoparticle. TrAD5-Ferritin was expressed by transfection of Expi293F cells and spontaneously self-assembled to form particles displaying eight trimeric gB-AD5 secreted into the culture supernatant. In contrast, trAD5-I5350A fusion was secreted as a homotrimer and only later assembled with I53-50.4BPT1 to form nanoparticles displaying twenty trimeric gB-AD5. (**Fig 3C and 3D**).

### Characterization of trAD5-nanoparticles structure-based vaccine

**Fig 4:**
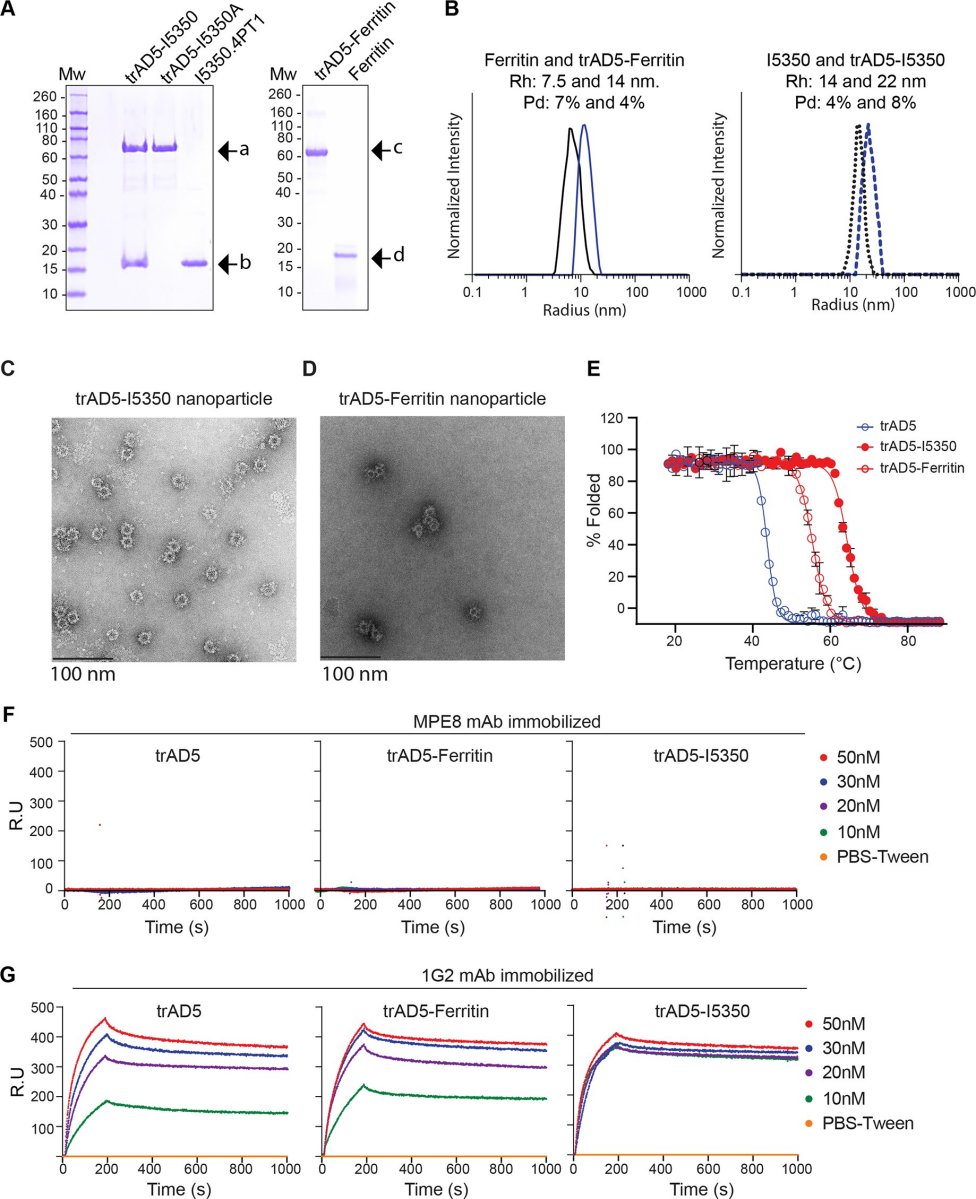
Characterization of trAD5-Ferritin and trAD5-I5350. (**A**) Reducing SDS-PAGE of SEC-purified components and nanoparticle immunogens. Arrow indicated specific protein (a is trAD5-I5350A fusion, b is I53-50.4PT1, c is trAD5-Ferritin and d is Ferritin). Molecular weight marker is indicated in kilodalton (kDa). (**B**) Dynamic Light Scattering of Ferritin, trAD5-Ferritin, I5350 and trAD5-I5350 nanoparticles. The hydrodynamic radius (Rh) and polydispersity (Pd) of each nanoparticle are indicated. (**C**) Images gallery of trAD5-I5350 and (**D**) trAD5-Ferritin. Scale bars indicates 100 nm. (**E**) Thermal denaturation of trAD5, trAD5-I5350 and trAD5-Ferritin. Percentage of denatured proteins was plotted as a function of temperature, and Tm (melting temperature) was calculated by Boltzmann regression curve fit. (**F**) Antigenic characterization of trAD5, trAD5-I5350 and trAD5-Ferritin nanoparticles by SPR with an irrelevant mAb (MPE8) used as control and (**G**) with 1G2 mAb, specific for AD5. The mAbs were immobilized on 5 channels of the chip, 3 for 1G2 and 2 for MPE8 and one channel was kept as blank surface with no antibody for use as a reference. Analyzed proteins (trAD5, trAD5-Ferritin and trAD5–I5350 nanoparticles), were injected at various concentrations (10 to 50nM) and interact specifically with 1G2. Data were processed using Proteon manager software and analyzed using Langmuir fitting. Each figure showed one representative result of three independent experiments.

To characterize these nanoparticles, we first examined the purity of both bare nanoparticle and trAD5-nanoparticle preparation by SDS-PAGE analysis (**Fig 4A**). As expected, bare Ferritin appeared as a single band of 20 kDa while trAD5-Ferritin migrated as a monomer of 65 kDa. The I5350 nanoparticle behaves as expected with the two subunits visible at 70 kDa and 18 kDa for trAD5-I5350A and I53-50.4PT1, respectively. To characterize the correct assembly of the particles, we measured the hydrodynamic radius (Rh) and *polydispersity* (Pd) index of all nanoparticles using Dynamic Light Scattering (DLS) (**Fig 4B)**. Bare Ferritin nanoparticles showed a Rh of 7.5nm and 14nm when trAD5 was displayed (**Fig 3C**). As expected, I5350 and trAD5-I5350 also appeared as single peaks with an Rh of 14nm, and 22 nm respectively (**Fig 3D**). These data were in agreement with the designed models, indicating that both preparations were aggregation-free and with the expected molecular weight. Finally, the overall structural validation was performed using negative-stain electron microscopy (EM) (**Fig 4C** and **4D**). We observed the regular icosahedral and octahedral symmetry for the bare I5350 and Ferritin nanoparticles, respectively. Using 2D averaging for the bare nanoparticles (**Figs 4B and S4A**), we could generate a low resolution map for the nanoparticles scaffold with a 3D reconstruction at 20Å and 19Å for the Ferritin and I5350 nanoparticle, respectively (**S4C** and **S4D Fig)**. However, we could not resolve trAD5 structure, since this domain is linked by a flexible glycine-serine linker (G_4_S) to the nanoparticle (**Fig 4C** and **4D**).

Next, we characterized the stability of the different antigens using circular dichroism (CD) thermal denaturation (**Fig 4E**). We analyzed the variation of ellipticity at 222 nm for trAD5 from 20°C to 90°C and we could calculate a Tm of ~45.5°C using Boltzmann regression fit. Interestingly, both nanoparticles conferred a stabilization effect, with a Tm of 58.2°C and 66°C for trAD5-Ferritin and trAD5-I5350 respectively. These results proved that fusion to hyper-stable nanoparticles significantly increase the overall antigen stability (**Fig 4E**). To evaluate if our vaccine candidates, displaying multivalent trAD5, were able to increase the relative affinity of the antigen *in vitro*, we decided to test BCR binding using Surface Plasmon Resonance (SPR). We evaluated the binding kinetic of the nanoparticle to the well-defined anti-AD5 mAb 1G2 [23] or anti-F-RSV mAb used as control [40]. Both mAbs were immobilized at 100nM on a GLM chip, and SPR measurement of the Kon, Koff and Kd for each antigen was performed. As expected, we could not detect any binding even of the AD5 design to MPE8 (**Fig 4F**). In contrast, we measured a Kon with 1G2 that was similar for trAD5, trAD5-Ferritin and trAD5-I5350 with 5.7x10^5^, 6.3x10^5^ and 7.5x10^5^ (1/Ms), respectively. On the other hand, the Koff was significantly lower for the nanoparticle constructs with 1x10^-6^ for trAD5-I5350 and 4x10^-5^ for trAD5-Ferritin compared to 1x10^-4^ (1/s) for trAD5 (**Fig 4G**). Our result indicates that once bound to the mAb, nanoparticles do not dissociate as fast as the soluble antigen, suggesting an increase in the relative affinity reflected by the avidity effect.

### Nanoparticles displaying trAD5 generate enhanced antibody levels and neutralization titers

**Fig 5:**
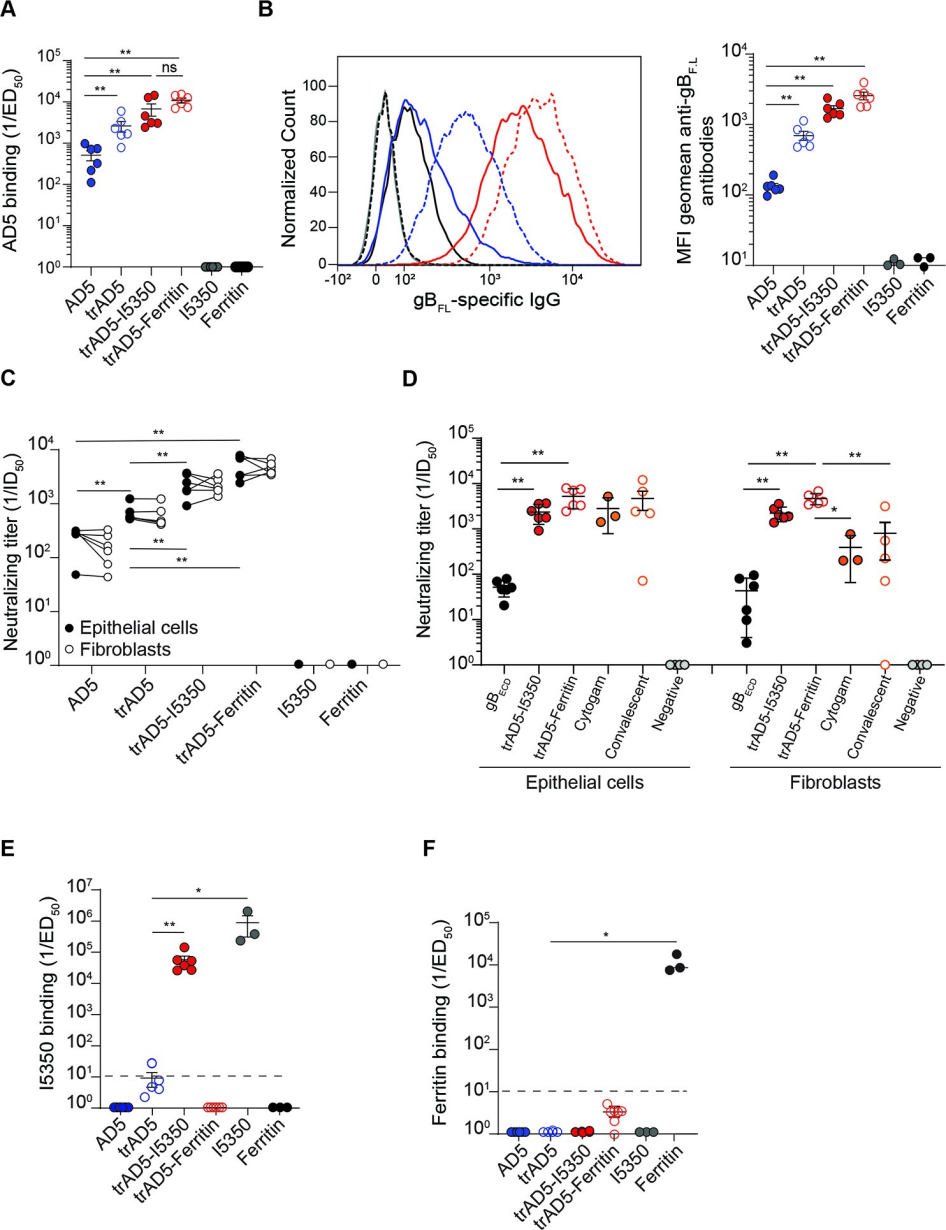
The trAD5-nanoparticles enhance gB immunogenicity in mice. (**A**) Inverse serum antibody binding titers (1/ED_50_) to AD5. Shown is the geometric mean. (**B**) Flow cytometry detection of gB_FL_-specific IgG antibodies in mice sera. Left panel show the average MFI of each individual mouse, shown in **S4B Fig** and right panel show the MFI geometric mean of each individual mouse. Shown are sera from AD5 (blue), trAD5 (open circle blue), trAD5-I5350 (red), trAD5-Ferritin (open red circle), I5350 (gray) and Ferritin (black) immunized mice. 1G2 control mAb is shown in black. Quantifications performed on the geometric mean of MFI+/-SD (right). (**C**) Inverse serum antibody neutralizing titers (1/ID_50_) measured on epithelial cells (ARPE-19, black) or fibroblasts (MRC-9, white). (**D**) Comparison of the neutralization titer (1/ID50) between sera from gB_ECD_ (black closed circle), trAD5-I5350 (red closed circle), trAD5-Ferritin (open red circle), Cytogam (orange closed circle) and convalescent sera (orange open circle). Shown in grey are titers of seronegative donors. (**E**) Inverse IgG serum antibody binding titers (1/ED_50_) to I5350, plotted as in A. (**F**) Inverse IgG serum antibody binding titers (1/ED_50_) to Ferritin, plotted as in A. Each figure shows one representative result of three independent experiment. Significance was calculated using Kruskal-Wallis + post hoc Mann-Whitney U test. Marked with (*) for p < 0.05, (**) for p < 0.01, and (***) for p < 0.001. Plotted are geometric means and error bars show SD of the geometric mean values.

To investigate the potential benefit of our designs, we decided to immunize BALB/c mice with soluble monomeric AD5, soluble trimeric AD5 (trAD5), trAD5-Ferritin and trAD5-I5350 nanoparticles. All immunogens were formulated with Ribi adjuvant for priming and boost I, while second boost was done without. Mice were immunized with 5μg of AD5 or trAD5 antigen (or 5μg of bare I5350 and Ferritin nanoparticles), using the same time schedule of the experiment described in **Fig 2**. As expected, immunization with bare Ferritin and I5350 nanoparticles did not induce any specific response against AD5 (**Fig 5A**), while sera from mice immunized with AD5, trAD5, trAD5-Ferritin and trAD5-I5350 nanoparticles elicited strong antibody responses (**Fig 5A**). We measured a binding titer (ED_50_) of 10^2.7^, 10^3.4^, 10^3.8^ and 10^4.1^ for AD5, trAD5, trAD5-I5350 and trAD5-Ferritin, respectively (**Fig 5A**). Interestingly, we noticed that trAD5 induced a binding titer that was 5–fold higher than the monomeric AD5, a result indicating that our trimeric design has been successful. Both nanoparticles displaying trAD5 on their surface induced an even more potent immune response, with a 12-fold (trAD5-I5350) and 25-fold (trAD5-Ferritin) higher antigen binding titer compared to the soluble AD5 (**Fig 5A**). To confirm this astonishing result, we decided to analyze the binding of mice sera to the full-length membrane anchored gB protein (gB_FL_). The gB_FL_ (Merlin strain) was transfected in Expi293F cells and its expression on cell membrane was confirmed by flow cytometry using specific mAbs binding to gB. We used mAb ITC52 (directed against AD1), mAb 6B4 (directed against AD2), mAb 7H3 (directed against AD4), mAb 1G2 (directed against AD5) as positive control and mAb 15D8 recognizing pUL128 as negative control (**S5A Fig**) [56,57]. Moreover, we also preformed staining on gHgL (Merlin strain) transfected Expi293F cells as an additional specificity control (**S5A Fig**). Expi293F cells expressing gB_FL_ were stained with sera of each individual mice (**S5B Fig**), and we observed an higher average geometric mean fluorescence intensity (MFI) with sera from mice immunized with trAD5-nanoparticles compared to sera derived from mice immunized with the soluble-particulate antigen (**Fig 5B**). All together, these results demonstrate that trAD5-nanoparticles are able to elicit higher antibody responses compared to the soluble AD5 antigen.

Next, we wanted to investigate if the higher binding titer translated also in a higher neutralization titer. We calculated the neutralization titer on both epithelial cells and fibroblast obtaining an ID_50_ of 2.81E+02, 6.9E+02, 2.36E+03 and 5.25E+03 (epithelial cells) and 1.77E+02, 6.2E+02, 2.24E+03 and 4.71E+03 (fibroblasts) for AD5, trAD5, trAD5-I5350 and trAD5-Ferritin respectively (**Fig 5C**). These results confirmed again the immunogenic superior properties of trAD5-nanoparticles compared to the soluble AD5, highlighted by a 10 and 20-fold higher neutralization titer on epithelial cells and 12 and 30-fold increase on fibroblast for the trAD5-I5350 and trAD5-Ferritin, respectively. To correlate our finding to other studies characterizing gB as a potential vaccine candidate, we compared the neutralization titers induced by our nanoparticles vaccines with titers induced by gB_ECD_ and the well-characterized benchmark Cytogam, an IgG solution containing a standardized amount of antibody to Cytomegalovirus and used in clinic [58]. We also add convalescent sera from seropositive and seronegative individuals. The comparison is not fully optimal, as Cytogam and human sera contain antibodies reactive against several HCMV proteins and glycoproteins. Nevertheless, we calculated a mean of neutralizing titers in ARPE-19 epithelial cells of 5.2E+01, 2.36E+03, 5.25E+03, 2.83E+03 and 4.68E+03 for gB_ECD,_ trAD5-I5350, trAD5-Ferritin, Cytogam and convalescent sera, respectively. A result clearly demonstrating the superiority of the nanoparticle platform in comparison to the soluble protein (**Fig 5D)**. Moreover, a similar result were obtained on MRC-9 fibroblast, with a mean neutralization titers of 4.3E+01, 2.24E+03, 4.7E+03, 3.2E+02 and 7.9E+02 for gB_ECD,_ trAD5-I5350, trAd5-Ferritin, Cytogam and convalescent sera, respectively (**Fig 5D)**. Taken together these results clearly illustrate the compelling effect of our domain mapping and nanoparticle formulation platform. Surprisingly, we observed that trAD5-I5350 nanoparticles, that possess a higher valency of antigen compared to trAD5-Ferritin nanoparticles, did not generate a higher antibody binding titer nor a better neutralizing titer. One observation that could explain these results is the higher titer of antibodies generated against the I5350 scaffold (with an ED_50_ 10^4.7^) (**Fig 5E**), compared to Ferritin nanoparticles which induce only an ED_50~_10^1^ against the nanoparticle (**Fig 5F**). Altogether, our results indicate that trAD5-nanoparticles boost the humoral immune response and strongly increase the sera neutralization activity between 50 and 100 folds based on the scaffold used and cell type investigated compared to the gB_ECD_ immunogen.

## Discussion

HCMV is a medically relevant human pathogen and a target for therapeutic intervention [59]. Despite HCMV eliciting a strong humoral and cellular immune response after natural infection, achievement of complete sterilizing immunity remains an unmet medical need. Several complexes are present on the virion envelope, the trimeric complex composed of gH, gL and gO glycoprotein subunits is necessary for infection of all cell types, including fibroblast, epithelial, and endothelial cells [60–63]. The pentameric complex composed of gH, gL and pUL128, pUL130, pUL131A subunits is further required for efficiently targeting HCMV to epithelial and endothelial cells. Upon mice immunization, the pentamer was shown to elicit extremely potent neutralizing antibodies (nAbs) directed against the pULs subunit and able to block cellular entry in epithelial and endothelial cells at picoMolar concentration [39]. Nevertheless, only the mAbs targeting gB homotrimer and gH/gL dimer proteins are able to block viral entry in all cell types, although these mAbs have a lower potency [39,47]. The abundant virion envelope glycoprotein B (gB) is a key target for vaccine development and was use in phase II clinical trials as HCMV vaccine [64]. Following both natural infection and recombinant gB vaccination, some gB-specific antibodies have been reported to have neutralizing activity, although the majority of them are non-neutralizing. Consequently, the gB vaccine showed only a modest efficacy in preventing primary viral infections [16,17]. Given the promising improvement of a recent gB candidate vaccine lacking its AD3 domain (22), we thought that a detailed analysis of the immunogenicity of the different gB antigenic domains would pave the way for new vaccine design. Our analysis, revealed some unexpected results. We observed that some murine AD1 antibodies could neutralize HCMV in presence of complement. It was previously reported that complement plays a role in antibody-mediated neutralization [16,49]. Recently, some rabbit mAbs targeting gB were reported, among them two mAbs, r272.7 and r210.4 exhibited neutralizing activity only in presence of complement [49]. Furthermore, the neutralization activity of both mAbs appeared to be associated with their epitope specificities. It is tempting to speculate that these mAbs were targeting AD1. The domain IV (AD1) is supposed to be in close proximity with the fusion peptide, both of them being located behind AD5, when gB is in its prefusion conformation [65]. It might be possible that mAbs targeting AD1 would recruit the complement and promote viral lysis or cell lysis of infected cell expressing gB on their surface. The mechanism of HCMV control by non-neutralizing mAbs is still under investigation, while antibody-dependent cellular cytotoxicity (ADCC) was demonstrated to be key in this mechanism [16]. It is conceivable that mAbs targeting AD1 and AD5 are especially important for ADCC. This hypothesis should be further investigated to understand the precise function of mAbs targeting different ADs in the promotion of ADCC and Antibody Dependent Cellular Phagocytosis (ADCP) for HCMV control in vaccinated individual.

Additionally, we demonstrated that AD5 is the main target of gB specific HCMV neutralizing antibodies. Interestingly, a recent publication corroborates this finding, demonstrating that syncytium formation could be significantly inhibited only by neutralizing mAbs targeting AD5 [66], other antibodies targeting different gB ADs were shown to block cell-to-cell spreading. However, this discovery is strongly correlating with AD5 being the driver of the neutralization response [67]. In the last decades, neutralizing antibodies targeting AD1, AD2 and AD4 have been characterized [30,32]. However, our single domain immunization strategy revealed that although these antigenic domains can be the target of some neutralizing antibodies, they mostly induce non-sterilizing immunity. The precise mechanism behind our observation is still not clearly identified. We can speculate that AD5 is a target of choice as it contains the predicted fusion loops [23,24]. It is also possible that antibody binding to AD5 impaired gB interaction with gHgL that forms the core machinery for HCMV fusogenic activity [14,15]. Moreover, the metastable prefusion conformation of gB in the native environment of HCMV virions was captured by cryoelectron tomography [65]. Using structures sub-tomographic averaging, the Hong Zhou group obtained structures at 21Å resolution and performed domain modeling indicating that the AD5 domain was not highly rearranged from pre- to post-fusion transition.

In this report, we used antigen discovery, computational modeling and generated a stable trimerized version of AD5 (trAD5). This viral antigen was further functionalized using the self-assembling nanoparticle methodology previously shown to enhance the immunogenicity of HIV [68], EBV [53], influenza [36] and RSV viruses [37]. Our computationally designed two-components and Ferritin nanomaterials are capable of scaffolding and stabilizing trAD5 and induce potent HCMV-neutralizing antibody responses far more superior than the soluble antigen. Although there is no formal correlate of protection from infection, several lines of evidence indicate that high levels of neutralizing antibodies protect against severe HCMV disease. We found that in mice, trAD5-nanoparticles induce roughly 10-20-fold higher levels of neutralizing antibodies during epithelial cells infection and 12-to 30-fold increase in fibroblast compare to soluble AD5. Overall, we provide an example of a general approach to modern vaccinology. We started from the analysis of the neutralizing antibody response elicited by a pathogen or a vaccine candidate to identify, engineer and produce a recombinant subunit vaccine capable of eliciting the most effective antibody response. The combination of “analytical vaccinology” [47] with rational computational protein design and structural biology is here demonstrated to deliver a new vaccine candidate that is more efficient than any previous candidate and induce antibody neutralizing titers that are similar to those produce by natural infection.

## Material and methods

### Ethics statement

Animal procedures were performed in accordance with the guidelines of the Swiss Federal Veterinary Office and after obtaining ethical approval from the Ufficio Veterinario Cantonale, Bellinzona, Switzerland (approval number TI-36-2018).

### Protein expression and purification

All DNA sequences were codon-optimized for expression in human cells and were based on the HCMV Merlin strain [69]. The following constructs, gB_ECD_, AD2, AD4, AD5, trAD5-I5350A, trAD5-Ferritin, Ferritin and I5350A were ordered from GenScript and cloned in the pcDNA3.1^+^ mammalian cell expression plasmid (Invitrogen). Of note, DNA for AD1 and I5350B.4PT1 were codon-optimized for bacterial cell expression and sub-cloned into the pET21a^+^ expression vector (Twist Bioscience). For protein purification purposes, all the genes encoded a C-Terminal TEV protease site, a hexaHistidine and twin-Strep Tag domains. All constructs were sequence verified and proteins produced by transfection of Expi293F cells using linear 25kDa polyethyleneimine (PEI; Polysciences). Transfected cells were maintained for 7 days at 37°C, 85% humidity, 8% CO_2_, with shaking at 135 rpm. The supernatant was loaded over one 5mL HisTrap Excel column (GE Healthcare) using an AKTA Pure system (GE Healthcare) and eluted with a linear gradient of 50 mM to 1 M imidazole (Sigma) in PBS pH 7.5. The IMAC eluate was purified by affinity chromatography on Strep-Tactin XT resin (IBA Lifesciences) and eluted with Buffer BXT (IBA Lifesciences). Next, Size Exclusion Chromatography (SEC) with a HiLoad 16/60 Superdex 200 prep grade GL column (GE Healthcare) run in PBS (Gibco), or PBS with 5% glycerol (Thermo Fisher Scientific) for trAD5-I5350A was performed. SEC-purified target proteins were snap frozen in liquid nitrogen and stored at -80°C.

For AD1 and I5350B.4PT1 expression plasmid was transformed into BL21Rosetta (DE3) E. coli cells (Novagen). Bacteria were grown in LB medium (Sigma) supplemented with 34 μg/L of chloramphenicol (Sigma) and 50 μg/L of ampicillin (Sigma) at 37°C, with shaking at 220 rpm, until an OD600 of 0.7 was reached. Protein expression was induced by addition of 1 mM isopropyl-thio-β-D-galactopyranoside (Sigma) and allowed to proceed for 3 h at 37°C, with shaking at 220 rpm, before cells were harvested by centrifugation. Cell pellets were suspended in extraction buffer (20 mM Tris-HCl, 0.5 M NaCl, 5 mM imidazole, 6 M guanidine hydrochloride, 1mM 2-mercaptoethanol pH 8.0), homogenized and obtained lysate was cleared by centrifugation and filtered through a 0.22 μm filter. The protein was purified from the filtered supernatant by IMAC via gravity column with cOmplete His-Tag Purification Resin (Roche) using extraction buffer as a wash and PBS supplemented with 1 M imidazole as elution. Peak fraction was concentrated in 5 kDa MWCO centrifugal filters, sterile filtered (0.22 μm) and applied to a Superdex 200 Increase 10/300 GL SEC column using PBS. The SEC-purified target protein was snap frozen in liquid nitrogen and stored at -80°C. The I5350A trimer and I5350B.4PT1 pentamer were expressed and purified as previously described [70].

### Design of trimeric AD5 and docking to I5350 and Ferritin nanoparticles

The crystal structure of gB (PDB ID 5C6T) was used as a template for building the trimeric AD5 (trAD5). The amino acids 131 to 347 were linked to the core domain (475 to 531) by a flexible G_4_S linker. The model was minimized using YASARA force field [54]. Connectivity was determined using the Average Degree filter in ROSETTA [71]. Images were rendered with PyMOL (Schrodinger, LLC).

### *In vitro* assembly of trAD5-I5350 and I5350

*In vitro* assembly was previously described [37]. Briefly, trAD5-I5350A trimer or I5350A trimer (in 25 mM Tris pH 8, 500 mM NaCl, 0.75% CHAPS) was first added to an Eppendorf tube to a final concentration of 10 μM in the *in vitro* assembly reaction. Assembly buffer (25 mM Tris pH 8, 250 mM NaCl, 5% glycerol) was then added to a volume of 1 mL minus the total volumes of the components. Finally, I53-50B.4PTI pentamer (in 25 mM Tris pH 8, 500 mM NaCl, 0.75% CHAPS) was added to the tube for a final concentration of 10 μM. Assemblies were incubated at 4°C for at least 1 h. Assembled nanoparticles were sterile filtered (0.22 μm) before subsequent purification by SEC using a Superdex 200 Increase 10/300 GL SEC column.

### Circular Dichroism (CD) spectroscopy and thermal denaturation

CD spectra from recombinant proteins (0.5 mg/mL in 10 mM NaPO_4_, pH 7.4) were recorded on a Chirascan spectropolarimeter (Applied Photophysics) over the wavelength range of 180 to 260 nm at a bandwidth of 1 nm, step size of 0.5 nm and 1 s per step. The spectra in the far-ultraviolet region required an average of five scans and were subtracted from blank spectra performed with a buffer. Measurements of thermal denaturation performed with a Tramp. of 1°C/min. CD spectra of the trAD5, trAD5-Ferritin, trAD5-I5350 at 0.5 mg/mL were acquired every 1°C increase, between 20°C and 90°C; percentage of denatured protein (loss of signal at 222 nm) was plotted as a function of temperature, and Tm (melting temperature) was calculated by Boltzmann sigmoidal nonlinear regression curve fit.

### Size Exclusion Chromatography (SEC)-HPLC analysis

For SEC-HPLC, 10μg of ADs or gB_ECD_ in PBS were separated with the Agilent 1100 HPLC machine using TSK-GEL G3000SW column (Tosoh, bed volume: 13 mL, void volume: 4.6 mL) with PBS as the mobile phase (flow rate: 1 mL/min). A universal solvent 2-μm filter (Agilent) was put between injector and column. Detection was performed using a Variable Wavelength Detector (Agilent) with ultraviolet absorption at 220 nm.

### Dynamic Light Scattering (DLS)

Triplicate measurements of 20 acquisitions each at 5 s per acquisition were taken on a DynaPro Nanostar instrument at 25°C in a 1 mL quartz cuvette (Wyatt Technology Corp) and using auto-attenuation of the laser. Increased viscosity due to the inclusion of 5% glycerol in the trAD5-I5350 nanoparticles was accounted for in the software.

### Surface Plasmon Resonance (SPR)

The experiments were performed at 25°C on a ProteON XPR-36 instrument (Bio-Rad Laboratories) in PBS and 0.05% Tween-20 (Sigma). The mAbs 1G2 [23] and MPE8 [40] were immobilized on a 3 and 2 channels respectively of a GLM sensor chip surface (Bio-Rad) through amine coupling at 100 nM and a blank surface with no protein was created under identical coupling conditions for use as a reference. Analyte proteins (trAD5, trAD5-I5350 and trAD5-Ferritin), were injected at a flow rate of 100 μL/min, at concentrations of 50, 30, 20 and 10 nM in different sensor channels. The data were processed using Proteon Manager Software and double-referenced by subtraction of the blank surface and buffer-only injection. K*on*, K*off*, and KD were calculated using Langmuir fitting.

### Negative stain electron microscopy

For the negative stain EM appearing in **Figs 4 and S4**, stock solutions of I5350, Ferritin, trAD5-I5350 and trAD5-Ferritin were diluted with PBS to an optimal concentration for automatic single particle acquisition. The samples were adsorbed to a glow-discharged carbon-coated copper grid 400mesh (EMS, Hatfield, PA, USA) washed with deionized water and stained with a solution of uranyl acetate 1% for 30 seconds. Observation was made using an F20 electron microscope (Thermo Fisher, Hillsboro, USA) operated at 200 kV. Digital images were collected using a direct detector camera Falcon III (Thermo Fisher, Hillsboro, USA) 4098 X 4098 pixels. Automatic data collection was performed using EPU software (Thermo Fisher, Hillsboro, USA) at a nominal magnification of 62,000X, corresponding to pixel size of 1.65 Å using a defocus range from -1 μm to -2.5μm. All images pre-processing, two-dimensional classification and three-dimensional processing was done using CryoSPARC software [72]. Particle images were extracted using a box size of 400 pixels with an effective pixel size of 1.65 Å. After 2 rounds of reference-free 2D classification, an initial model with icosahedral or octahedral symmetry were generated from 2D class averages. The refined map were used as a reference for one additional round of 3D refinement to obtain the final map at an estimated resolution of 20Å for I5350 (based on 8467 particles) and 19Å Ferritin (based on 5467 particles). Visualization, fitting and final rendering of figures was performed using UCSF Chimera. EM maps were deposited to EMD with the following entry EMD-11398 for I5350 and EMD-11400 for Ferritin.

### Mice immunizations

Female, 6–9 week-old BALB/c mice were obtained from Charles River (Italy). All proteins were dialyzed in PBS and then formulated by a 1:1 mix ratio with Ribi Adjuvant (Sigma) for priming and boost one, according to the manufacturer’s instructions. Mice were immunized subcutaneously (s.c.) with a total protein dose of 5 μg or corresponding to 5μg of the trAD5 antigen on day 0 and 21 and 35. Mice were bled on day 45 and recovered sera were used to measure binding and neutralization titers.

### Enzyme-Linked Immunosorbent Assay (ELISA)

Enzyme-Linked Immunosorbent Assay (ELISA) was used to determine binding of sera and mAbs to the different proteins. Maxisorp (Nunc) ELISA plates were coated overnight at 4°C with 3 μg/mL of antigen in PBS. Plates were blocked with 1% w/v solution of Bovine Serum Albumin (BSA; Sigma) in PBS for 1 h at room temperature (R.T.). Serial dilutions of human mAbs or mice sera were added to the plates and, after washing, antibody binding was revealed using a goat anti-human IgG antibody coupled to alkaline phosphatase (AP) (Jackson Immunoresearch) for human mAbs or with goat anti-mouse IgG antibody coupled to AP (Jackson Immunoresearch) for murine sera. Plates were then washed, substrate (p-NPP, Sigma) added and absorbance read at 405 nm.

### Cells and virus

Expi293F cells were grown in Expi293 expression media, cultured at 37°C, 85% humidity, 8% CO_2_, with shaking at 135 rpm. MRC-9, a human embryonic lung fibroblast cell line (ATCC CCL-212), was grown in Eagle’s Minimal Essential Medium (EMEM) with Earle’s salts (Sigma) + GlutaMAX (Thermo Fisher Scientific) supplemented with 10% Fetal Bovine Serum (FBS) plus 100 IU/mL Penicillin/Streptomycin (Thermo Fisher Scientific) and cultured at 37°C, and 5% CO_2_. ARPE-19, a retinal pigment epithelial cell line (ATCC CRL-2302), was grown in Dulbecco's Modified Eagle Medium/Nutrient Mixture F-12 (DMEM/F-12) (Thermo Fisher Scientific) + GlutaMAX (Thermo Fisher Scientific) supplemented with 10% Fetal Bovine Serum (FBS) plus 100 IU/mL Penicillin/Streptomycin (Thermo Fisher Scientific) and cultured at 37°C, and 5% CO_2_. All cell lines were confirmed to be free of Mycoplasma. HCMV endothelial cell adapted VR1814 was provided by Dr. Daniele Lilleri from UOC Microbiologia e Virologia. Fondazione IRCCS Policlinico San Matteo. Pavia, Italy.

### Virus micro-neutralization

Serial dilutions of sera were pre-incubated with HCMV clinical isolate (strain VR1814) for 1 h at 37°C and added to confluent monolayers of ARPE-19 or MRC-9 cells cultured in 384-well flat-bottom plates (MOI of 1). Serial dilution (1:3) were used for micro neutralization assay, with the first dilution at 1: 50. Cytogam (CSL Behring) was tested in a similar way with a first dilution 1:50, also it is worth noting that the immunoglobulin (Ig) content in mouse serum, as approximately 10 time lower as previously described [73]. After 48 h, cells were fixed with 5% acetic acid in methanol and then stained with 0.5 μg/mL of mouse anti-pp72 antibody (Clone 6E1.Santa Cruz Biotechnology, SC-69834). Cells were then incubated with 1 μg/mL of a goat F(ab)2 anti-mouse IgG (H+L) AlexaFluor 594-conjugated (Thermo Fisher Scientific) and counterstained with DAPI. Images were acquired on ImageXpress Micro Confocal bioimaging system (Molecular Devices LLC). The percentage of infected cells was automatically calculated by Imagexpress software. Dose-response curves were generated by plotting the relative infected cells against sera dilutions. The sera dilution causing inhibition of 50% of infection (ID_50_) was calculated by nonlinear regression with Prism 7 (GraphPad Software). For neutralization assay, in presence of rabbit complement (Sigma, S7764), we used 5% complement in a final volume of 200μl, plates were cultured for 2 days [74]. Convalescent sera were obtained from Dr. Daniele Lilleri.

### Sera immunodepletion

Mice sera (100 μL diluted to 1 mL in PBS) was injected into a HisTrap HP column (GE Healthcare) saturated with His-tagged AD5 antigen (50 mg/mL). Sera was left in the column for 1 h at R.T. Depleted sera were recovered by isocratic elution with PBS and further used for binding assays. Bounds antibodies were recovered by elution with 0.1 M glycine at pH 2.9.

### Murine B cell clones generation and screening

BALB/c mice immunized three times with the recombinant gB_ECD_ protein, were intravenously (i.v.) injected with a 5μg of antigen formulated in PBS. Mice were sacrificed on day 5 and spleens were harvested. Splenocyte cells were cultivated on feeder layer in presence of TLR9 agonist (ODN 1826, Invivogen). Cell suspension was distributed in 96-well round-bottom plates at 3 x 10^4^ cell/well. After one week, supernatants from B cell clones were screened for the presence of IgG and HCMV gB, AD1, AD2, AD4 and AD5-specific antibodies by ELISA. Briefly, maxisorp (Nunc) ELISA plates were coated overnight at 4°C with 3 μg/mL of antigen. Plates were blocked with 1% w/v solution of Bovine Serum Albumin (BSA; Sigma) in PBS. Supernatants were added to the plates and antibody binding was revealed using a goat anti-mouse IgG antibody coupled to AP (Jackson Immunoresearch). Plates were then washed, substrate (p-NPP, Sigma) added and absorbance read at 405 nm.

### Flow cytometry staining

Flow Cytometry assay were used to determine binding of sera to the gHgL as control and full-length gB protein, containing the transmembrane domain and AD3. The full-length gB, gH and gL glycoprotein (all from Merlin strain), were ordered from GenScripts and cloned in the pcDNA3.1^+^ mammalian expression plasmid (ThermoFisher) by transient transfection using PEI (Polyscience). Transfected cells were harvested after 3 days washed twice with PBS+2% FBS and incubated in PBS + 0.5% BSA + 2 mM EDTA with mice sera or the indicated specific human monoclonal antibodies or anti-pULs (15D8) and non-immunized mouse sera for isotype control at 2 μg/ml for 30 min on ice. After two washes, cells were incubated with goat anti-mouse IgG (H+L) Alexa Fluor 647 conjugated (Thermo Fisher Scientific) or goat anti-human IgG (H+L) Alexa Fluor 488 conjugated (Thermo Fisher Scientific) secondary antibodies at 2 μg/ml for 30 min on ice. Dead cells were excluded from counting by staining with 7-aminoactinomycin D (7-AAD; BioLegend). Samples were acquired with a FACS Fortessa (BD Biosciences) flow cytometer. Analysis was performed with FlowJo software (TreeStar).

### Statistical analysis

Statistical parameters including the exact value of n, the definition of center, dispersion, and precision measures (geometric mean ± SD) and statistical significance are reported in the Figures and Figure Legends. Data were judged to be statistically significant when p < 0.05. In figures, statistical significance is calculated using the two-tailed non-parametric Mann-Whitney U test for two groups’ comparison or Kruskall–Wallis test (and Dunn’s posttest) when three or more groups were compared when three or more groups were compared. Analyses were performed with PRISM 7 (GraphPad Software).

## Supporting information

S1 Fig Control of serum binding titers in mice immunized with gB_ECD_ and ADs. Inverse IgG serum antibody binding titers (1/ED_50_) to F-RSV as control. Panel showed one representative result of three independent experiments. Significance was calculated using Kruskal-Wallis + post hoc Mann-Whitney U test. Marked with (*) for p < 0.05, (**) for p < 0.01, and (***) for p < 0.001. Plotted are geometric means and Error bars show SD of the geometric mean values.
(TIF)

S2 Fig Circular Dichroism spectra of gB extracellular domain (gB_ECD_) and Antigenic Domains (ADs). Far-UV spectra for gB_ECD_ and ADs (0.5 mg/mL) were recorded over the wavelength range of 180–260 nm. Shown in black is gB_ECD_, in orange AD1, in grey AD2, in green AD4 and in blue AD5. The percentage of secondary structure is shown on the bottom right panel.
(TIF)

S3 Fig Serum binding and neutralizing titers in mice immunized with gB_ECD_ and ADs. (**A**) Inverse IgG serum antibody binding titers (1/ED_50_) to each AD. Error bars show SD of the geometric mean values. (**B**) Inverse IgG serum antibody binding titers (1/ED_50_) to F-RSV. Error bars show SEM of the geometric mean values. (**C**) Inverse IgG serum antibody neutralizing titers (1/ID50) measured on ARPE-19 epithelial cells (black circles without complement and white circles with complement). (**D**) Inverse IgG serum antibody neutralizing titers (1/ID_50_) measured on MRC-9 fibroblasts (black circles without complement and white circles with complement). Each assay was repeated two times. Plotted are geometric means and Error bars show SD of the geometric mean values.
(TIF)

S4 Fig Negative stain EM of nanoparticles. (**A)** Images gallery of bare I5350 and Ferritin (**B**) with 2D averaging. Scale bars indicates 100 nm. (**C**) Single-particle electron microscopy 3D reconstruction of bare I5350 at 20Å (EMD-11398) and (**D**) bare Ferritin at 19Å (EMD-11400).
(TIF)

S5 Fig Flow cytometry detection of gB_FL_ on Expi293F transfected cells. (**A**) Cell surface staining with anti-gB specific IgG antibodies, ITC52 binds to AD1, 6B4 binds to AD2, 7H3 binds to AD4 and 1G2 binds AD5. The 15D8 mAb (anti-pUL128) was use as negative control (top panel). Bottom panel shown cell surface staining on cells transfected with gHgL as negative control. (**B**) Staining of Expi293F cells, transfected with gB_FL,_ with each individual serum from immunized mice. Panels are organized by antigen used for each plot. Shown in red is pooled sera from non-immunized control mice.
(TIF)

## References

[1] Kenneson A, Cannon MJ. Review and meta-analysis of the epidemiology of congenital cytomegalovirus (CMV) infection. Rev Med Virol. 2007;17(4):253–76. Epub 2007/06/21. 10.1002/rmv.535 . 17579921

[2] Cannon MJ, Schmid DS, Hyde TB. Review of cytomegalovirus seroprevalence and demographic characteristics associated with infection. Rev Med Virol. 2010;20(4):202–13. Epub 2010/06/22. 10.1002/rmv.655 . 20564615

[3] Ross SA, Boppana SB. Congenital cytomegalovirus infection: outcome and diagnosis. Seminars in pediatric infectious diseases. 2005;16(1):44–9. Epub 2005/02/03. 10.1053/j.spid.2004.09.011 . 15685149

[4] Britt W. Manifestations of human cytomegalovirus infection: proposed mechanisms of acute and chronic disease. Current topics in microbiology and immunology. 2008;325:417–70. Epub 2008/07/22. 10.1007/978-3-540-77349-8_23 . 18637519

[5] Legendre C, Pascual M. Improving outcomes for solid-organ transplant recipients at risk from cytomegalovirus infection: late-onset disease and indirect consequences. Clin Infect Dis. 2008;46(5):732–40. Epub 2008/01/29. 10.1086/527397 . 18220478

[6] Krishna BA, Wills MR, Sinclair JH. Advances in the treatment of cytomegalovirus. British Medical Bulletin. 2019;131(1):5–17. 10.1093/bmb/ldz031 31580403 PMC6821982

[7] Revello MG, Lazzarotto T, Guerra B, Spinillo A, Ferrazzi E, Kustermann A, et al A randomized trial of hyperimmune globulin to prevent congenital cytomegalovirus. The New England journal of medicine. 2014;370(14):1316–26. Epub 2014/04/04. 10.1056/NEJMoa1310214 . 24693891

[8] Arvin AM, Fast P, Myers M, Plotkin S, Rabinovich R. Vaccine development to prevent cytomegalovirus disease: report from the National Vaccine Advisory Committee. Clin Infect Dis. 2004;39(2):233–9. Epub 2004/08/13. 10.1086/421999 . 15307033

[9] Bernstein DI. Congenital Cytomegalovirus: a "Now" Problem-No Really, Now. Clinical and vaccine immunology: CVI. 2017;24(1). Epub 2016/11/01. 10.1128/CVI.00491-16 27795304 PMC5216425

[10] Thompson KM, Gellin BG, Hinman AR, Orenstein WA. The National Vaccine Advisory Committee at 30: Impact and opportunity. Vaccine. 2018;36(11):1330–44. 10.1016/j.vaccine.2018.01.068 . 29422369 PMC7115546

[11] Connolly SA, Jardetzky TS, Longnecker R. The structural basis of herpesvirus entry. Nature reviews Microbiology. 2020 Epub 2020/10/23. 10.1038/s41579-020-00448-w . 33087881 PMC8579738

[12] Nguyen CC, Kamil JP. Pathogen at the Gates: Human Cytomegalovirus Entry and Cell Tropism. Viruses. 2018;10(12). Epub 2018/12/14. 10.3390/v10120704 30544948 PMC6316194

[13] Malito E, Chandramouli S, Carfi A. From recognition to execution-the HCMV Pentamer from receptor binding to fusion triggering. Current opinion in virology. 2018;31:43–51. Epub 2018/06/06. 10.1016/j.coviro.2018.05.004 . 29866439

[14] Vanarsdall AL, Howard PW, Wisner TW, Johnson DC. Human Cytomegalovirus gH/gL Forms a Stable Complex with the Fusion Protein gB in Virions. PLoS Pathog. 2016;12(4):e1005564 Epub 2016/04/16. 10.1371/journal.ppat.1005564 27082872 PMC4833381

[15] Vanarsdall AL, Ryckman BJ, Chase MC, Johnson DC. Human cytomegalovirus glycoproteins gB and gH/gL mediate epithelial cell-cell fusion when expressed either in cis or in trans. J Virol. 2008;82(23):11837–50. Epub 2008/09/26. 10.1128/JVI.01623-08 18815310 PMC2583677

[16] Nelson CS, Huffman T, Jenks JA, Cisneros de la Rosa E, Xie G, Vandergrift N, et al HCMV glycoprotein B subunit vaccine efficacy mediated by nonneutralizing antibody effector functions. Proc Natl Acad Sci U S A. 2018;115(24):6267–72. Epub 2018/05/02. 10.1073/pnas.1800177115 29712861 PMC6004431

[17] Baraniak I, Kropff B, Ambrose L, McIntosh M, McLean GR, Pichon S, et al Protection from cytomegalovirus viremia following glycoprotein B vaccination is not dependent on neutralizing antibodies. Proc Natl Acad Sci U S A. 2018;115(24):6273–8. Epub 2018/04/25. 10.1073/pnas.1800224115 29686064 PMC6004462

[18] Pass RF, Zhang C, Evans A, Simpson T, Andrews W, Huang ML, et al Vaccine prevention of maternal cytomegalovirus infection. The New England journal of medicine. 2009;360(12):1191–9. Epub 2009/03/20. 10.1056/NEJMoa0804749 19297572 PMC2753425

[19] Vietzen H, Gorzer I, Honsig C, Jaksch P, Puchhammer-Stockl E. HCMV-Specific Antibody Response And Development Of ADCC Against HCMV After Lung Transplantation. J Infect Dis. 2020 Epub 2020/03/12. 10.1093/infdis/jiaa097 . 32157310

[20] Nelson CS, Jenks JA, Pardi N, Goodwin M, Roark H, Edwards W, et al Human Cytomegalovirus Glycoprotein B Nucleoside-Modified mRNA Vaccine Elicits Antibody Responses with Greater Durability and Breadth than MF59-Adjuvanted gB Protein Immunization. J Virol. 2020;94(9). Epub 2020/02/14. 10.1128/JVI.00186-20 32051265 PMC7163130

[21] Baraniak I, Gomes AC, Sodi I, Langstone T, Rothwell E, Atkinson C, et al Seronegative patients vaccinated with cytomegalovirus gB-MF59 vaccine have evidence of neutralising antibody responses against gB early post-transplantation. EBioMedicine. 2019;50:45–54. Epub 2019/11/19. 10.1016/j.ebiom.2019.11.005 31735553 PMC6921368

[22] Schleiss MR. Recombinant cytomegalovirus glycoprotein B vaccine: Rethinking the immunological basis of protection. Proc Natl Acad Sci U S A. 2018;115(24):6110–2. Epub 2018/06/08. 10.1073/pnas.1806420115 29875141 PMC6004476

[23] Chandramouli S, Ciferri C, Nikitin PA, Calo S, Gerrein R, Balabanis K, et al Structure of HCMV glycoprotein B in the postfusion conformation bound to a neutralizing human antibody. Nature communications. 2015;6:8176 Epub 2015/09/15. 10.1038/ncomms9176 26365435 PMC4579600

[24] Burke HG, Heldwein EE. Crystal Structure of the Human Cytomegalovirus Glycoprotein B. PLoS Pathog. 2015;11(10):e1005227 10.1371/journal.ppat.1005227 26484870 PMC4617298

[25] Schoppel K, Kropff B, Schmidt C, Vornhagen R, Mach M. The humoral immune response against human cytomegalovirus is characterized by a delayed synthesis of glycoprotein-specific antibodies. J Infect Dis. 1997;175(3):533–44. Epub 1997/03/01. 10.1093/infdis/175.3.533 . 9041323

[26] Meyer H, Masuho Y, Mach M. The gp116 of the gp58/116 complex of human cytomegalovirus represents the amino-terminal part of the precursor molecule and contains a neutralizing epitope. J Gen Virol. 1990;71 (Pt 10):2443–50. Epub 1990/10/01. 10.1099/0022-1317-71-10-2443 . 1700066

[27] Meyer H, Sundqvist VA, Pereira L, Mach M. Glycoprotein gp116 of human cytomegalovirus contains epitopes for strain-common and strain-specific antibodies. J Gen Virol. 1992;73 (Pt 9):2375–83. Epub 1992/09/01. 10.1099/0022-1317-73-9-2375 . 1383409

[28] Kniess N, Mach M, Fay J, Britt WJ. Distribution of linear antigenic sites on glycoprotein gp55 of human cytomegalovirus. J Virol. 1991;65(1):138–46. Epub 1991/01/01. 10.1128/JVI.65.1.138-146.1991 1702157 PMC240498

[29] Silvestri M, Sundqvist VA, Rudén U, Wahren B. Characterization of a major antigenic region on gp55 of human cytomegalovirus. J Gen Virol. 1991;72 (Pt 12):3017–23. Epub 1991/12/01. 10.1099/0022-1317-72-12-3017 . 1662693

[30] Potzsch S, Spindler N, Wiegers AK, Fisch T, Rucker P, Sticht H, et al B cell repertoire analysis identifies new antigenic domains on glycoprotein B of human cytomegalovirus which are target of neutralizing antibodies. PLoS Pathog. 2011;7(8):e1002172 Epub 2011/08/20. 10.1371/journal.ppat.1002172 21852946 PMC3154849

[31] Spindler N, Diestel U, Stump JD, Wiegers AK, Winkler TH, Sticht H, et al Structural basis for the recognition of human cytomegalovirus glycoprotein B by a neutralizing human antibody. PLoS Pathog. 2014;10(10):e1004377 Epub 2014/10/10. 10.1371/journal.ppat.1004377 25299639 PMC4192593

[32] Spindler N, Rücker P, Pötzsch S, Diestel U, Sticht H, Martin-Parras L, et al Characterization of a discontinuous neutralizing epitope on glycoprotein B of human cytomegalovirus. J Virol. 2013;87(16):8927–39. Epub 2013/06/07. 10.1128/JVI.00434-13 23740990 PMC3754028

[33] Bachmann MF, Jennings GT. Vaccine delivery: a matter of size, geometry, kinetics and molecular patterns. Nature reviews Immunology. 2010;10(11):787–96. Epub 2010/10/16. 10.1038/nri2868 . 20948547

[34] Irvine DJ, Swartz MA, Szeto GL. Engineering synthetic vaccines using cues from natural immunity. Nature materials. 2013;12(11):978–90. Epub 2013/10/24. 10.1038/nmat3775 24150416 PMC3928825

[35] Perotti M, Perez L. Virus-Like Particles and Nanoparticles for Vaccine Development against HCMV. Viruses. 2019;12(1). Epub 2020/01/08. 10.3390/v12010035 31905677 PMC7019358

[36] Kanekiyo M, Joyce MG, Gillespie RA, Gallagher JR, Andrews SF, Yassine HM, et al Mosaic nanoparticle display of diverse influenza virus hemagglutinins elicits broad B cell responses. Nature immunology. 2019;20(3):362–72. Epub 2019/02/12. 10.1038/s41590-018-0305-x 30742080 PMC6380945

[37] Marcandalli J, Fiala B, Ols S, Perotti M, de van der Schueren W, Snijder J, et al Induction of Potent Neutralizing Antibody Responses by a Designed Protein Nanoparticle Vaccine for Respiratory Syncytial Virus. Cell. 2019;176(6):1420–31 e17. Epub 2019/03/09. 10.1016/j.cell.2019.01.046 30849373 PMC6424820

[38] Baraniak I, Kropff B, McLean GR, Pichon S, Piras-Douce F, Milne RSB, et al Epitope-Specific Humoral Responses to Human Cytomegalovirus Glycoprotein-B Vaccine With MF59: Anti-AD2 Levels Correlate With Protection From Viremia. J Infect Dis. 2018;217(12):1907–17. Epub 2018/03/13. 10.1093/infdis/jiy102 29528415 PMC5972559

[39] Macagno A, Bernasconi NL, Vanzetta F, Dander E, Sarasini A, Revello MG, et al Isolation of human monoclonal antibodies that potently neutralize human cytomegalovirus infection by targeting different epitopes on the gH/gL/UL128-131A complex. J Virol. 2010;84(2):1005–13. Epub 2009/11/06. 10.1128/JVI.01809-09 19889756 PMC2798344

[40] Corti D, Bianchi S, Vanzetta F, Minola A, Perez L, Agatic G, et al Cross-neutralization of four paramyxoviruses by a human monoclonal antibody. Nature. 2013;501(7467):439–43. 10.1038/nature12442 . 23955151

[41] Kelly SM, Jess TJ, Price NC. How to study proteins by circular dichroism. Biochim Biophys Acta. 2005;1751(2):119–39. Epub 2005/07/20. 10.1016/j.bbapap.2005.06.005 . 16027053

[42] Hill CP, Anderson DH, Wesson L, DeGrado WF, Eisenberg D. Crystal structure of alpha 1: implications for protein design. Science. 1990;249(4968):543–6. Epub 1990/08/03. 10.1126/science.2382133 . 2382133

[43] Marcos E, Chidyausiku TM, McShan AC, Evangelidis T, Nerli S, Carter L, et al De novo design of a non-local β-sheet protein with high stability and accuracy. Nature structural & molecular biology. 2018;25(11):1028–34. Epub 2018/10/31. 10.1038/s41594-018-0141-6 30374087 PMC6219906

[44] Yoon HS, Hajduk PJ, Petros AM, Olejniczak ET, Meadows RP, Fesik SW. Solution structure of a pleckstrin-homology domain. Nature. 1994;369(6482):672–5. Epub 1994/06/23. 10.1038/369672a0 . 8208296

[45] Tomai MA, Solem LE, Johnson AG, Ribi E. The adjuvant properties of a nontoxic monophosphoryl lipid A in hyporesponsive and aging mice. J Biol Response Mod. 1987;6(2):99–107. Epub 1987/04/01. . 3585413

[46] Ozorowski G, Cupo A, Golabek M, LoPiccolo M, Ketas TA, Cavallary M, et al Effects of Adjuvants on HIV-1 Envelope Glycoprotein SOSIP Trimers In Vitro. J Virol. 2018;92(13). Epub 2018/04/20. 10.1128/JVI.00381-18 29669838 PMC6002727

[47] Kabanova A, Perez L, Lilleri D, Marcandalli J, Agatic G, Becattini S, et al Antibody-driven design of a human cytomegalovirus gHgLpUL128L subunit vaccine that selectively elicits potent neutralizing antibodies. Proc Natl Acad Sci U S A. 2014;111(50):17965–70. Epub 2014/12/03. 10.1073/pnas.1415310111 25453106 PMC4273412

[48] Gomes AC, Griffiths PD, Reeves MB. The Humoral Immune Response Against the gB Vaccine: Lessons Learnt from Protection in Solid Organ Transplantation. Vaccines. 2019;7(3). Epub 2019/07/20. 10.3390/vaccines7030067 . 31319553 PMC6789498

[49] Li F, Freed DC, Tang A, Rustandi RR, Troutman MC, Espeseth AS, et al Complement enhances in vitro neutralizing potency of antibodies to human cytomegalovirus glycoprotein B (gB) and immune sera induced by gB/MF59 vaccination. NPJ vaccines. 2017;2:36 Epub 2017/12/22. 10.1038/s41541-017-0038-0 29263890 PMC5730571

[50] Foglierini M, Marcandalli J, Perez L. HCMV Envelope Glycoprotein Diversity Demystified. Front Microbiol. 2019;10:1005 Epub 2019/06/04. 10.3389/fmicb.2019.01005 31156572 PMC6529531

[51] Speckner A, Glykofrydes D, Ohlin M, Mach M. Antigenic domain 1 of human cytomegalovirus glycoprotein B induces a multitude of different antibodies which, when combined, results in incomplete virus neutralization. J Gen Virol. 1999;80 (Pt 8):2183–91. Epub 1999/08/31. 10.1099/0022-1317-80-8-2183 . 10466818

[52] Lopez-Sagaseta J, Malito E, Rappuoli R, Bottomley MJ. Self-assembling protein nanoparticles in the design of vaccines. Comput Struct Biotechnol J. 2016;14:58–68. Epub 2016/02/11. 10.1016/j.csbj.2015.11.001 26862374 PMC4706605

[53] Kanekiyo M, Bu W, Joyce MG, Meng G, Whittle JR, Baxa U, et al Rational Design of an Epstein-Barr Virus Vaccine Targeting the Receptor-Binding Site. Cell. 2015;162(5):1090–100. Epub 2015/08/19. 10.1016/j.cell.2015.07.043 26279189 PMC4757492

[54] Krieger E, Joo K, Lee J, Lee J, Raman S, Thompson J, et al Improving physical realism, stereochemistry, and side-chain accuracy in homology modeling: Four approaches that performed well in CASP8. Proteins. 2009;77 Suppl 9(Suppl 9):114–22. Epub 2009/09/22. 10.1002/prot.22570 19768677 PMC2922016

[55] Meier S, Güthe S, Kiefhaber T, Grzesiek S. Foldon, the natural trimerization domain of T4 fibritin, dissociates into a monomeric A-state form containing a stable beta-hairpin: atomic details of trimer dissociation and local beta-hairpin stability from residual dipolar couplings. Journal of molecular biology. 2004;344(4):1051–69. Epub 2004/11/17. 10.1016/j.jmb.2004.09.079 . 15544812

[56] Schoppel K, Hassfurther E, Britt W, Ohlin M, Borrebaeck CA, Mach M. Antibodies specific for the antigenic domain 1 of glycoprotein B (gpUL55) of human cytomegalovirus bind to different substructures. Virology. 1996;216(1):133–45. Epub 1996/02/01. 10.1006/viro.1996.0040 . 8614980

[57] Martinez-Martin N, Marcandalli J, Huang CS, Arthur CP, Perotti M, Foglierini M, et al An Unbiased Screen for Human Cytomegalovirus Identifies Neuropilin-2 as a Central Viral Receptor. Cell. 2018;174(5):1158–71 e19. 10.1016/j.cell.2018.06.028 . 30057110

[58] Doron S, Ruthazer R, Werner BG, Rabson A, Snydman DR. Hypogammaglobulinemia in liver transplant recipients: incidence, timing, risk factors, and outcomes. Transplantation. 2006;81(5):697–703. Epub 2006/03/15. 10.1097/01.tp.0000180531.66518.9e . 16534471

[59] Plotkin SA, Boppana SB. Vaccination against the human cytomegalovirus. Vaccine. 2018 Epub 2018/04/07. 10.1016/j.vaccine.2018.02.089 . 29622379 PMC6892274

[60] Kabanova A, Marcandalli J, Zhou T, Bianchi S, Baxa U, Tsybovsky Y, et al Platelet-derived growth factor-alpha receptor is the cellular receptor for human cytomegalovirus gHgLgO trimer. Nature microbiology. 2016;1(8):16082 10.1038/nmicrobiol.2016.82 27573107 PMC4918640

[61] Wille PT, Knoche AJ, Nelson JA, Jarvis MA, Johnson DC. A human cytomegalovirus gO-null mutant fails to incorporate gH/gL into the virion envelope and is unable to enter fibroblasts and epithelial and endothelial cells. J Virol. 2010;84(5):2585–96. Epub 2009/12/25. 10.1128/JVI.02249-09 20032184 PMC2820920

[62] Zhou M, Lanchy JM, Ryckman BJ. Human Cytomegalovirus gH/gL/gO Promotes the Fusion Step of Entry into All Cell Types, whereas gH/gL/UL128-131 Broadens Virus Tropism through a Distinct Mechanism. J Virol. 2015;89(17):8999–9009. Epub 2015/06/19. 10.1128/JVI.01325-15 26085146 PMC4524070

[63] Wu K, Oberstein A, Wang W, Shenk T. Role of PDGF receptor-α during human cytomegalovirus entry into fibroblasts. Proc Natl Acad Sci U S A. 2018;115(42):E9889–e98. Epub 2018/10/03. 10.1073/pnas.1806305115 30275317 PMC6196492

[64] Nelson CS, Baraniak I, Lilleri D, Reeves MB, Griffiths PD, Permar SR. Immune Correlates of Protection Against Human Cytomegalovirus Acquisition, Replication, and Disease. J Infect Dis. 2020;221(Supplement_1):S45–s59. Epub 2020/03/07. 10.1093/infdis/jiz428 32134477 PMC7057792

[65] Si Z, Zhang J, Shivakoti S, Atanasov I, Tao CL, Hui WH, et al Different functional states of fusion protein gB revealed on human cytomegalovirus by cryo electron tomography with Volta phase plate. PLoS Pathog. 2018;14(12):e1007452 Epub 2018/12/07. 10.1371/journal.ppat.1007452 30507948 PMC6307773

[66] Reuter N, Kropff B, Schneiderbanger JK, Alt M, Krawczyk A, Sinzger C, et al Cell fusion induced by a fusion-active form of human cytomegalovirus glycoprotein B is inhibited by antibodies directed at AD-5 in the ectodomain of gB. Journal of Virology. 2020:JVI.01276-20 10.1128/JVI.01276-20 32641474 PMC7459561

[67] Bialas KM, Westreich D, Cisneros de la Rosa E, Nelson CS, Kauvar LM, Fu TM, et al Maternal Antibody Responses and Nonprimary Congenital Cytomegalovirus Infection of HIV-1-Exposed Infants. J Infect Dis. 2016;214(12):1916–23. Epub 2016/12/08. 10.1093/infdis/jiw487 27923951 PMC5142097

[68] He L, de Val N, Morris CD, Vora N, Thinnes TC, Kong L, et al Presenting native-like trimeric HIV-1 antigens with self-assembling nanoparticles. Nature communications. 2016;7:12041 Epub 2016/06/29. 10.1038/ncomms12041 27349934 PMC4931238

[69] Dolan A, Cunningham C, Hector RD, Hassan-Walker AF, Lee L, Addison C, et al Genetic content of wild-type human cytomegalovirus. J Gen Virol. 2004;85(Pt 5):1301–12. Epub 2004/04/24. 10.1099/vir.0.79888-0 . 15105547

[70] Bale JB, Gonen S, Liu Y, Sheffler W, Ellis D, Thomas C, et al Accurate design of megadalton-scale two-component icosahedral protein complexes. Science. 2016;353(6297):389–94. Epub 2016/07/28. 10.1126/science.aaf8818 27463675 PMC5485857

[71] Leman JK, Weitzner BD, Lewis SM, Adolf-Bryfogle J, Alam N, Alford RF, et al Macromolecular modeling and design in Rosetta: recent methods and frameworks. Nature methods. 2020;17(7):665–80. 10.1038/s41592-020-0848-2 32483333 PMC7603796

[72] Punjani A, Rubinstein JL, Fleet DJ, Brubaker MA. cryoSPARC: algorithms for rapid unsupervised cryo-EM structure determination. Nature methods. 2017;14(3):290–6. Epub 2017/02/07. 10.1038/nmeth.4169 . 28165473

[73] Natsuume-Sakai S, Motonishi K, Migita S. Quantitative estimations of five classes of immunoglobulin in inbred mouse strains. Immunology. 1977;32(6):861–6. Epub 1977/06/01. 885588 PMC1445447

[74] Jomaa M, Yuste J, Paton JC, Jones C, Dougan G, Brown JS. Antibodies to the iron uptake ABC transporter lipoproteins PiaA and PiuA promote opsonophagocytosis of Streptococcus pneumoniae. Infection and immunity. 2005;73(10):6852–9. Epub 2005/09/24. 10.1128/IAI.73.10.6852-6859.2005 16177364 PMC1230898

