# Supplementary figures and images for: Rationally designed Human Cytomegalovirus gB nanoparticle vaccine with improved immunogenicity
Source: PLoS Pathog. 2020 Dec 28;16(12):e1009169. doi: 10.1371/journal.ppat.1009169 (PMC7794029; doi:10.1371/journal.ppat.1009169)

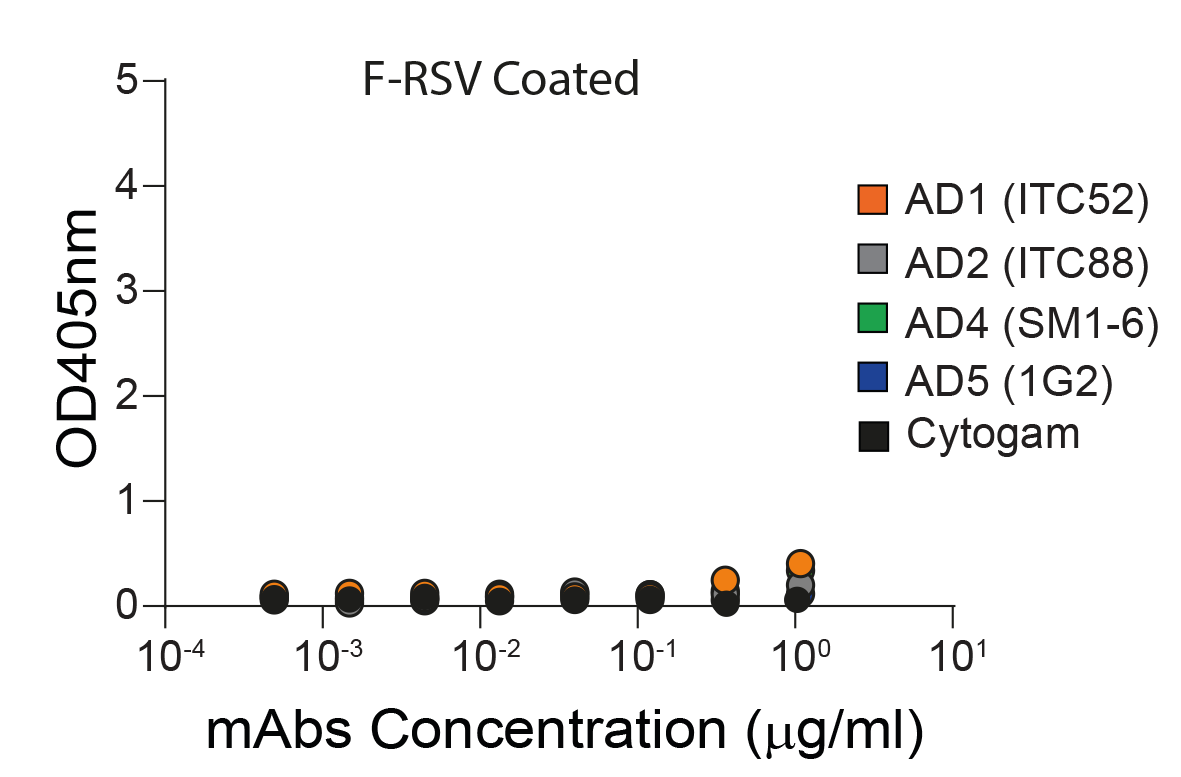

Supplement: S1 Fig — Inverse IgG serum antibody binding titers (1/ED50) to F-RSV as control. Panel showed one representative result of three independent experiments. Significance was calculated using Kruskal-Wallis + post hoc Mann-Whitney U test. Marked with (*) for p < 0.05, (**) for p < 0.01, and (***) for p < 0.001. Plotted are geometric means and Error bars show SD of the geometric mean values. (TIF) [file ppat.1009169.s001.tif]

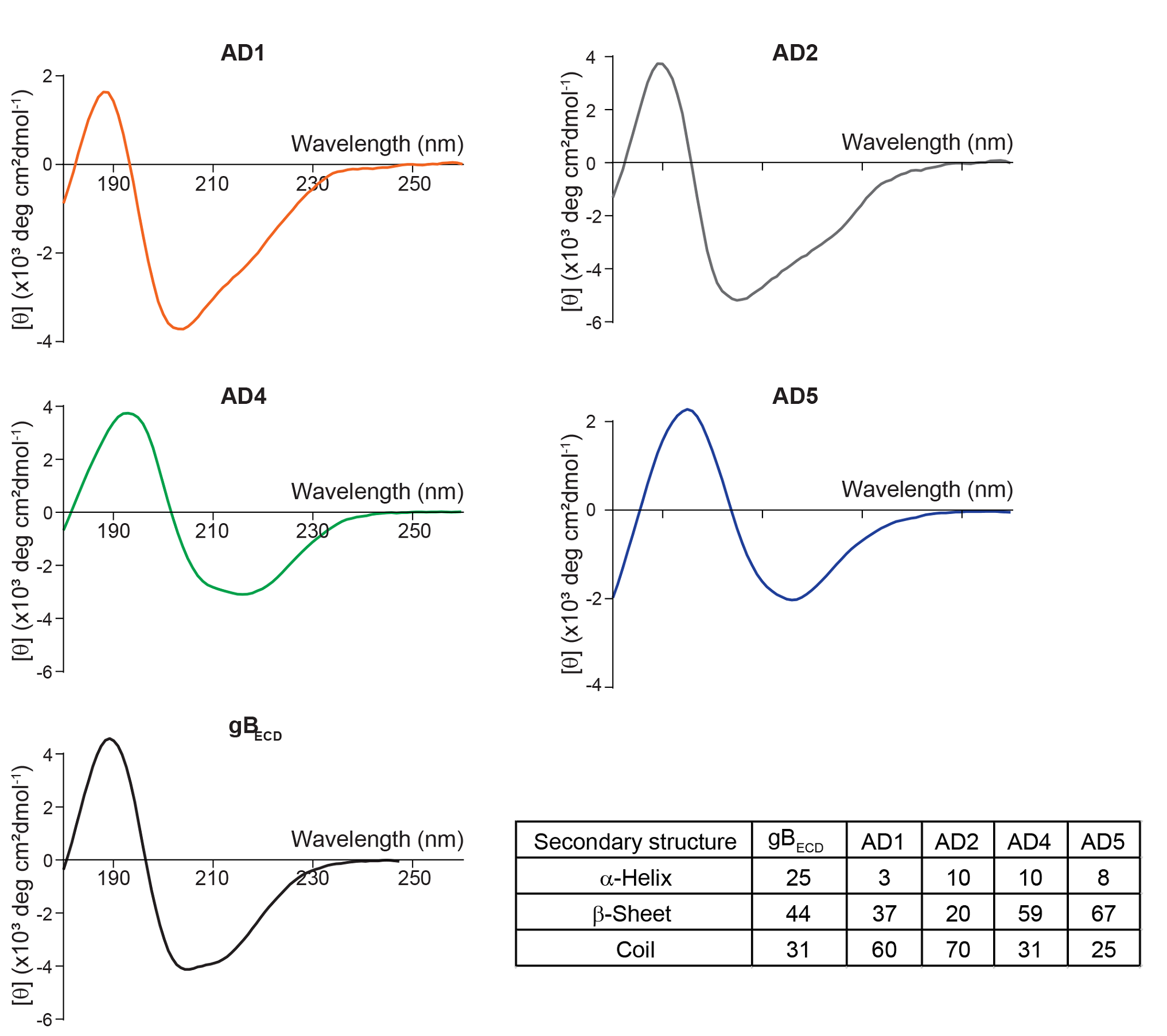

Supplement: S2 Fig — Far-UV spectra for gBECD and ADs (0.5 mg/mL) were recorded over the wavelength range of 180–260 nm. Shown in black is gBECD, in orange AD1, in grey AD2, in green AD4 and in blue AD5. The percentage of secondary structure is shown on the bottom right panel. (TIF) [file ppat.1009169.s002.tif]

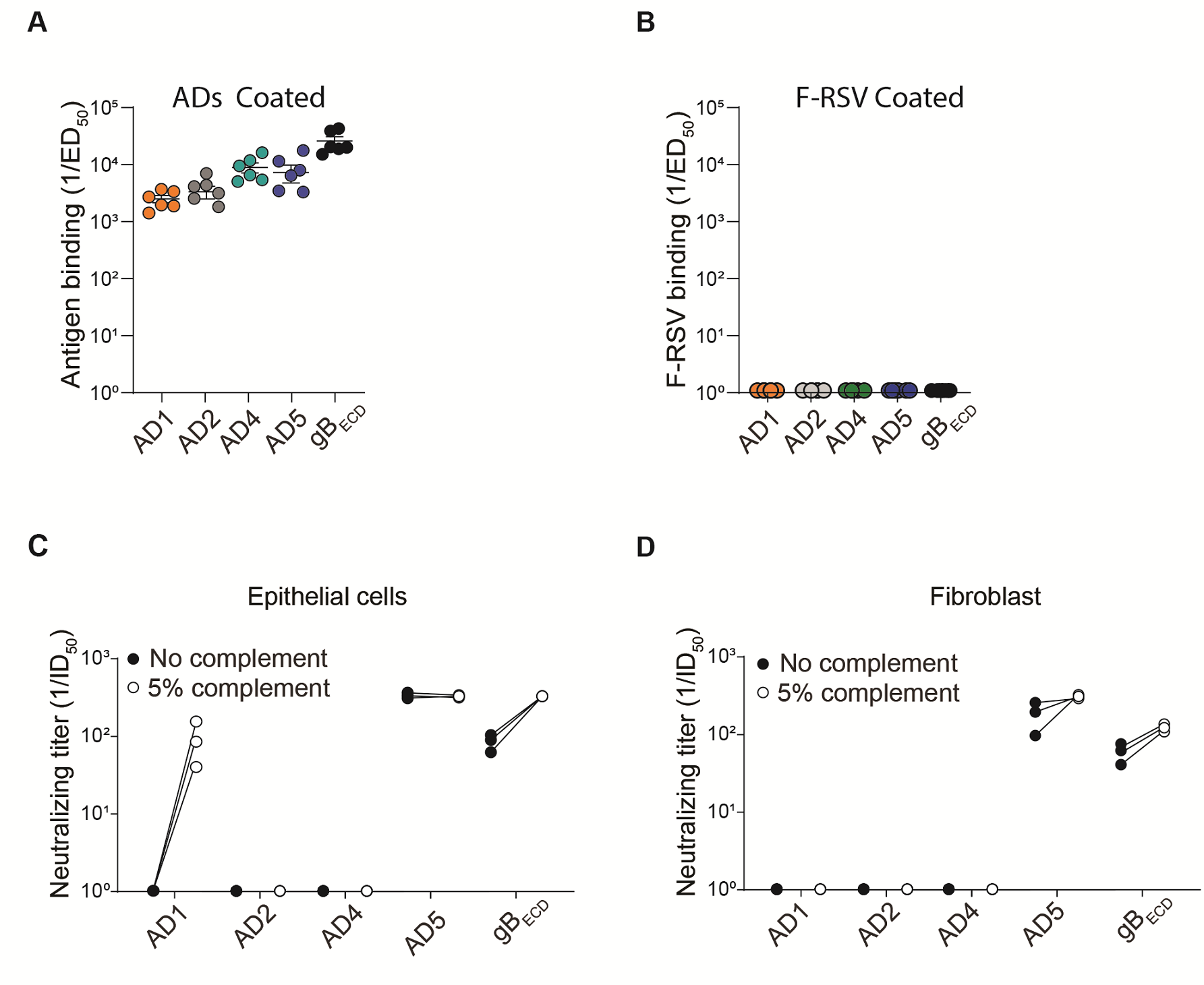

Supplement: S3 Fig — (A) Inverse IgG serum antibody binding titers (1/ED50) to each AD. Error bars show SD of the geometric mean values. (B) Inverse IgG serum antibody binding titers (1/ED50) to F-RSV. Error bars show SEM of the geometric mean values. (C) Inverse IgG serum antibody neutralizing titers (1/ID50) measured on ARPE-19 epithelial cells (black circles without complement and white circles with complement). (D) Inverse IgG serum antibody neutralizing titers (1/ID50) measured on MRC-9 fibroblasts (black circles without complement and white circles with complement). Each assay was repeated two times. Plotted are geometric means and Error bars show SD of the geometric mean values. (TIF) [file ppat.1009169.s003.tif]

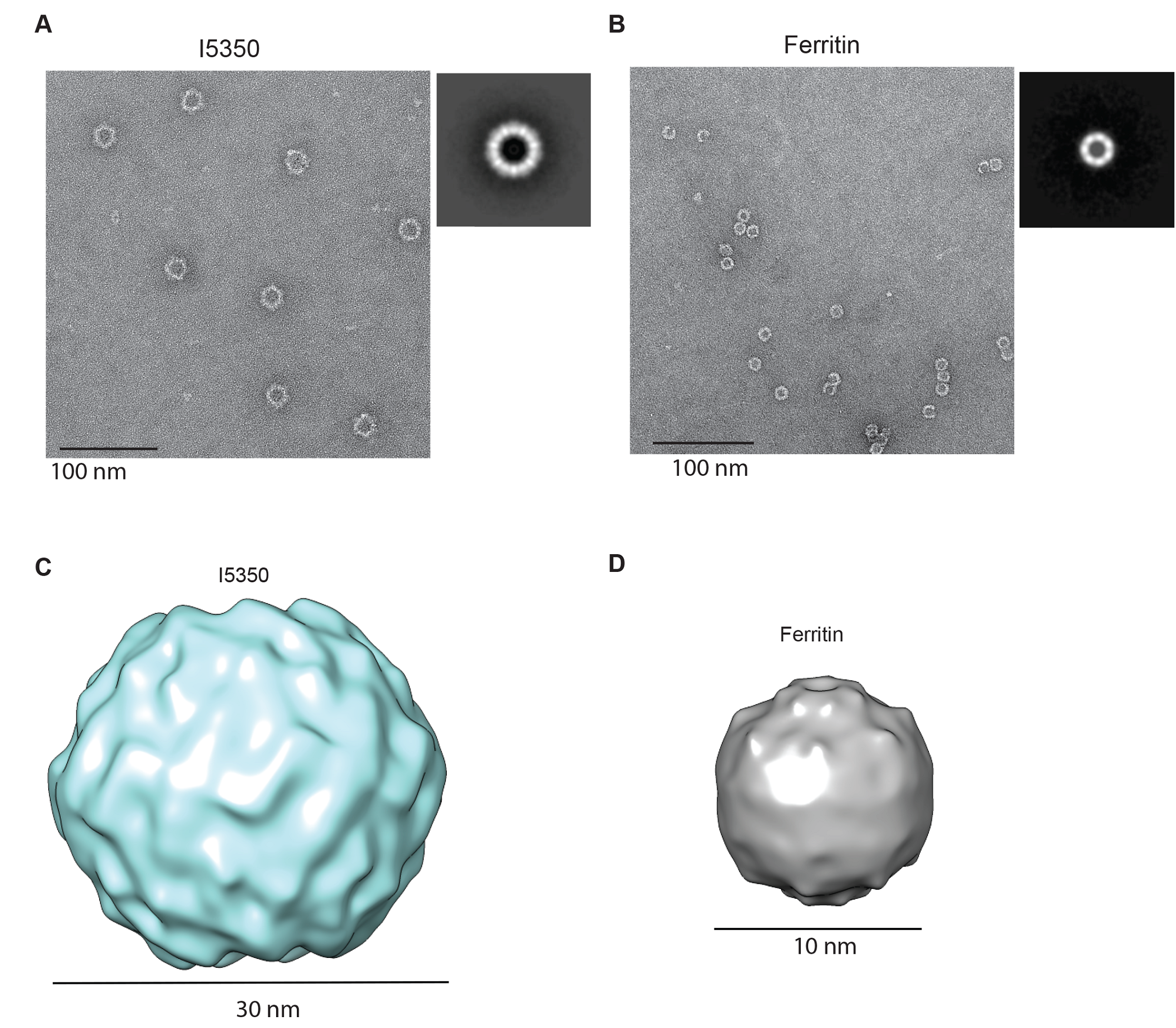

Supplement: S4 Fig — (A) Images gallery of bare I5350 and Ferritin (B) with 2D averaging. Scale bars indicates 100 nm. (C) Single-particle electron microscopy 3D reconstruction of bare I5350 at 20Å (EMD-11398) and (D) bare Ferritin at 19Å (EMD-11400). (TIF) [file ppat.1009169.s004.tif]

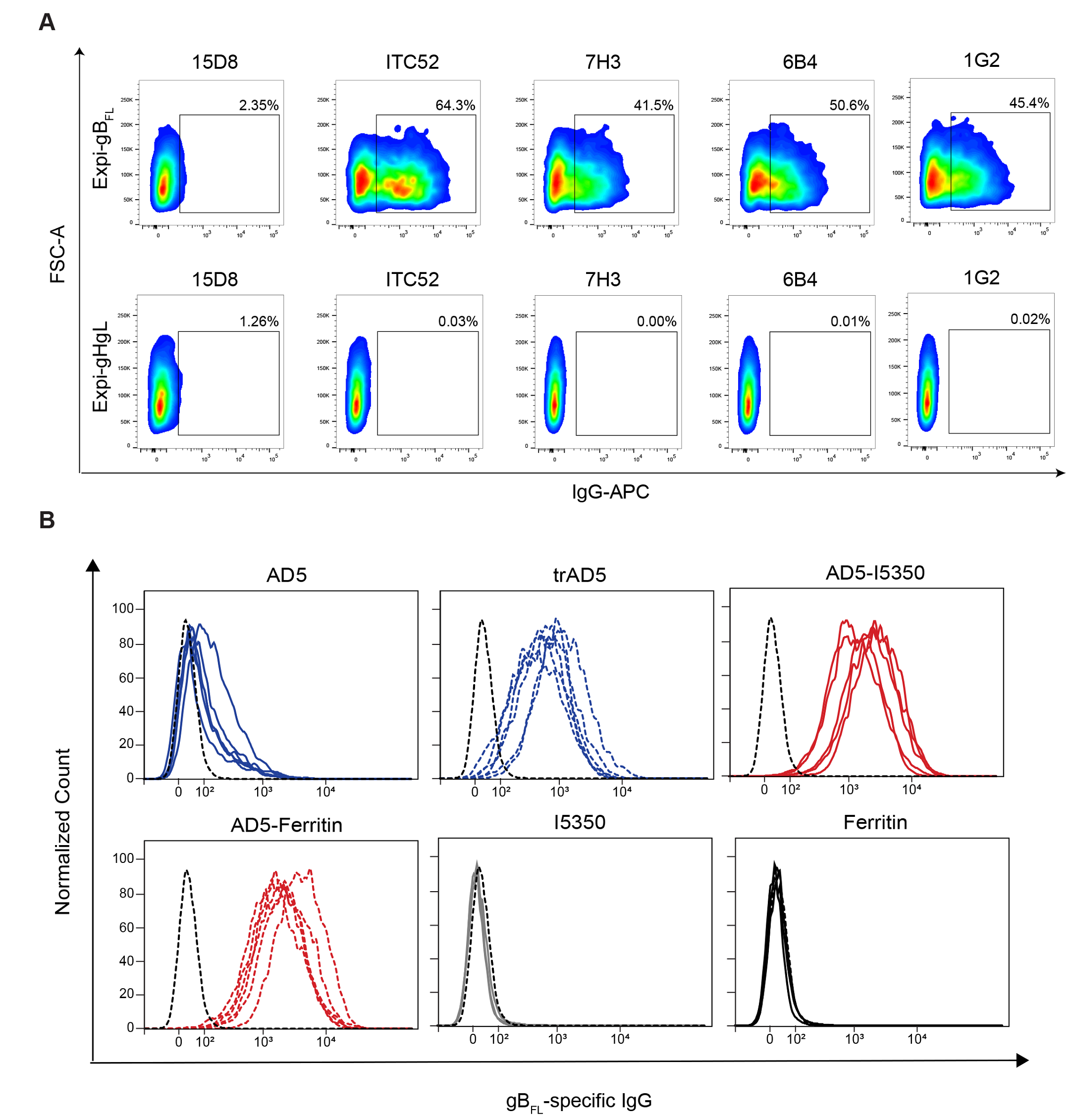

Supplement: S5 Fig — (A) Cell surface staining with anti-gB specific IgG antibodies, ITC52 binds to AD1, 6B4 binds to AD2, 7H3 binds to AD4 and 1G2 binds AD5. The 15D8 mAb (anti-pUL128) was use as negative control (top panel). Bottom panel shown cell surface staining on cells transfected with gHgL as negative control. (B) Staining of Expi293F cells, transfected with gBFL, with each individual serum from immunized mice. Panels are organized by antigen used for each plot. Shown in red is pooled sera from non-immunized control mice. (TIF) [file ppat.1009169.s005.tif]
